# PRMT5 inhibition sensitizes B-cell lymphoma cells to ferroptosis

**DOI:** 10.1038/s41375-026-02932-3

**Published:** 2026-04-17

**Authors:** Yunxia Liu, Ruoyu Chen, Xiaoyue Gao, Fen Zhu, Qinyu Ni, Paul D. Bates, Sunny Wu, Zhuoyan Zai, Victoria A. Obernberger, Kavinu Weerawardhene, Taylor K. Tourdot, Sophie Petta, Madison J. Conyers, Christian M. Capitini, Lixin Rui

**Affiliations:** 1 Department of Medicine, University of Wisconsin School of Medicine and Public Health, Madison, WI USA; 2 Carbone Cancer Center, University of Wisconsin School of Medicine and Public Health, Madison, WI USA; 3 Department of Pediatrics, University of Wisconsin School of Medicine and Public Health, Madison, WI USA

**Keywords:** Cancer metabolism, Haematological cancer

## Abstract

Protein arginine methyltransferase 5 (PRMT5) is overexpressed in B-cell lymphomas, including diffuse large B-cell lymphoma (DLBCL) and mantle cell lymphoma (MCL). While PRMT5 is known to regulate multiple oncogenic pathways, including PI3K-AKT signaling, its role in lipid metabolism and ferroptosis, a regulated, iron-dependent cell death driven by lipid peroxidation, remains poorly understood. Here, we identify a novel role for PRMT5 in suppressing ferroptosis in DLBCL and MCL cells through upregulation of SLC7A11, which imports cystine for glutathione (GSH) biosynthesis. This effect is mediated by the AKT-MYC-ATF5 signaling axis. ATF5, a MYC-regulated transcription factor overexpressed in these lymphomas, induces SLC7A11 expression. In addition, ATF5 promotes the expression of ATF4, another key regulator of the ferroptotic response, which forms heterodimers with ATF5 to further reinforce this regulatory network. PRMT5 inhibition sensitizes lymphoma cells to ferroptosis inducers such as dimethyl fumarate (DMF), an electrophile that irreversibly depletes GSH via succination. Notably, combined treatment with the PRMT5 inhibitor GSK3326595 and DMF synergistically enhances anti-tumor activity in a patient-derived xenograft (PDX) model. These findings reveal a previously unrecognized PRMT5-ATF5-SLC7A11 axis that drives ferroptosis resistance in B-cell lymphomas and provide a strong rationale for targeting PRMT5 to potentiate ferroptosis-based therapies in relapsed or refractory disease.

## Introduction

Non-Hodgkin lymphoma (NHL) is a heterogeneous group of malignancies, comprising over two dozen diagnostic entities, each with distinct molecular and genetic profiles, as well as varying pathological courses and clinical features [1]. Diffuse large B-cell lymphoma (DLBCL) is the most common form of B-cell NHL, accounting for 30-40% of lymphoma cases [1]. It has two main subtypes: germinal center B cell-like (GCB) and activated B cell-like (ABC) [2]. DLBCL has also been classified into up to seven distinct subgroups based on clustering of genetic alterations, enhancing the prediction of responses to first-line and targeted therapies [3, 4]. Despite advances in molecular classification, one-third of DLBCL patients relapse from or do not respond to first-line immunochemotherapy, which includes rituximab, cyclophosphamide, doxorubicin, vincristine, and prednisone (R-CHOP) [5–7]. Salvage treatments such as second-line chemotherapies (e.g., DHAP, ICE), targeted therapies (e.g., BTK inhibitors, CAR T-cell therapy), or stem cell transplantation are effective in fewer than 30% to 50% of these relapsed/refractory patients [8–12]. Mantle cell lymphoma (MCL) is a rare type of B-cell NHL that makes up about 6% of cases and is currently considered incurable [13, 14]. A hallmark of MCL is the t(11;14)(q13;q32) translocation, which juxtaposes the cyclin D1 proto-oncogene (CCND1) with the immunoglobulin heavy chain gene (IgH) and results in the constitutive expression of CCND1 [14]. There is currently no universally accepted standard of care for MCL. In addition to immunochemotherapy such as R-CHOP, targeted therapeutic strategies have been widely employed in its management. These include single or combination therapies with BTK inhibitors (e.g., ibrutinib, zanubrutinib, acalabrutinib, pirtobrutinib) [15–18], the BCL2 inhibitor venetoclax [19, 20], and CAR T-cell therapy [21, 22]. Although response rates to these therapies are usually high, the vast majority of patients still do not achieve long-term survival.

PRMT5, which regulates gene expression by symmetric dimethylation of histones and non-histone target proteins, is overexpressed and plays a pathogenic role in various solid tumors and hematological malignancies [23–27]. Our recent study has demonstrated that PRMT5 expression is upregulated in DLBCL and MCL [28]. PRMT5 overexpression promotes cell proliferation and survival in these lymphoma cells through multiple mechanisms, such as activating the PI3K-AKT pathway, MYC target genes and lipid metabolic reprogramming [28–30]. PRMT5 directly methylates all three members of the AKT family [31, 32], and MYC is a downstream target of AKT signaling [28]. Targeting PRMT5 with specific inhibitors has revealed anti-tumor effects in preclinical studies [27–29, 33]. However, a recent Phase 1 trial reported that the use of a PRMT5 inhibitor alone shows limited efficacy in solid tumors and NHL, including DLBCL and MCL [34].

Ferroptosis is an iron-dependent form of regulated cell death triggered by the accumulation of lipid peroxides on cellular membranes [35, 36]. Ferroptosis can be prevented by different cellular defense systems including glutathione peroxidase 4 (GPX4), solute carrier family 7 member 11 (SLC7A11 or xCT) [37] and ferroptosis suppressor protein 1 (FSP1 or AIFM2). GPX4 uses glutathione (GSH) as its cofactor to quench lipid peroxidation, while SLC7A11 imports cystine for GSH biosynthesis [37, 38]. AIFM2 inhibits ferroptosis independently of GPX4 by reducing ubiquinone (CoQ) to ubiquinol (CoQH2) at the plasma membrane, which functions as a lipophilic radical-trapping antioxidant, effectively capturing lipid peroxyl radicals [37, 38]. Cancer cells have evolved to upregulate the SLC7A11/GSH/GPX4 axis to avoid ferroptosis and promote their progression [37, 39, 40]. Targeting ferroptosis has emerged as a potential therapeutic strategy for cancer treatment [38, 39]. Recent preclinical studies have demonstrated that dimethyl fumarate (DMF), an FDA-approved drug for the treatment of multiple sclerosis and psoriasis, induces ferroptosis and synergizes with a ferroptosis inducer or a BRD4 inhibitor to inhibit the growth of DLBCL cells [41, 42]. Mechanistically, DMF acts as an electrophile that irreversibly depletes cellular GSH through a process termed succination, thereby impairing GPX4’s function and promoting lipid peroxidation [41, 42]. BRD4 inhibition with specific inhibitors reduces AIFM2 expression in DLBCL cells [41].

In this study, we demonstrate that PRMT5 is involved in ferroptosis in DLBCL and MCL. PRMT5 upregulates SLC7A11 expression through the transcription factor ATF5, whose expression is induced by the PI3K-AKT-MYC axis, a PRMT5 downstream signaling pathway. Genetic and pharmacological inhibition of PRMT5 with small guide RNA (sgRNA) or a specific inhibitor enhances DMF-mediated ferroptosis and cooperatively inhibits tumor growth in cell line- and patient-derived xenograft models.

## Materials and Methods

(See Supplemental Materials and Methods for details).

### Cell lines and culture conditions

All diffuse large B-cell lymphoma (DLBCL) and mantle cell lymphoma (MCL) cell lines were cultured in RPMI 1640 medium (Corning) supplemented with 10% FBS (Cytiva HyClone), 2 mM GlutaMax™ (Gibco), 1 mM sodium pyruvate (Cytiva HyClone), and 1% penicillin/streptomycin (Lonza). Primary MCL cells were maintained in RPMI 1640 medium supplemented with 20% FBS, with all other culture conditions identical to those used for cell lines. All cells were maintained at 37 °C in a humidified incubator with 5% CO₂. All cell lines were authenticated by STR profiling and routinely tested negative for mycoplasma contamination.

### Lipid peroxidation assay

Following the indicated treatments, 1 × 10⁶ cells per condition were exposed to the specified concentrations of dimethyl fumarate (DMF) or solvent control for 3 hours. Cells were then washed once with pre-warmed Hank’s Balanced Salt Solution (HBSS) and incubated with 1 μM BODIPY™ 581/591 C11 (Invitrogen) in HBSS for 20 min at 37 °C. After staining, cells were washed twice with HBSS and immediately analyzed by flow cytometry. Just prior to acquisition, 10 ng/μL DAPI was added to each sample to exclude non-viable cells. Fluorescence was measured using a ThermoFisher Attune flow cytometer, and data were analyzed using FlowJo software (version 10.0).

### MCL patient-derived xenograft model

Primary MCL samples used in this study were the same as those described in our recent studies [43, 44]. To establish the initial PDX model, 2 × 10⁶ MCL-4 cells were resuspended in 0.1 mL PBS and injected into the tail vein of sublethally irradiated (2 Gy) NOD scid γc⁻/⁻ (NSG) mice. After 4–6 weeks, spleens and livers were harvested to obtain single-cell suspensions of the tumor. Tumor cells derived from spleen and liver sources were separately inoculated into the flanks of NSG mice (5 × 10⁶ cells per mouse) to establish subcutaneous xenograft models. Mice were randomized into treatment groups receiving vehicle, DMF, GSK3326595, or a combination of both, at doses indicated in the main text or figure legends. MCL-4 cells were virally transduced to express luciferase. Bioluminescent imaging was performed using the Lago X imaging system (Spectral Instruments Imaging) at the indicated time points. Mice were anesthetized and injected intraperitoneally with 150 mg/kg D-luciferin (30 mg/mL in PBS; Gold Biotechnology), and images were acquired 15 min post-injection. Total photon flux (photons/second) was quantified for each mouse within defined regions of interest. Endpoint analyses were conducted 4 weeks after tumor cell injection.

### Statistical analysis

A two-tailed Student’s *t*-test was used to assess significant differences between two groups. One-way or two-way analysis of variance (ANOVA) was performed for comparisons among three or more groups. Survival analyses were conducted using the log-rank test. Data are presented as mean ± standard deviation (SD) or standard error of the mean (SEM), as indicated. Statistical significance was indicated as follows: *P < 0.05, **P < 0.01, ***P < 0.001, and ****P < 0.0001. No samples, animals, or biological replicates were excluded from the analyses. No predefined exclusion criteria were applied.

## Results

### PRMT5 inhibition promotes lipid peroxidation and enhances DMF-mediated ferroptosis

**Fig. 1:**
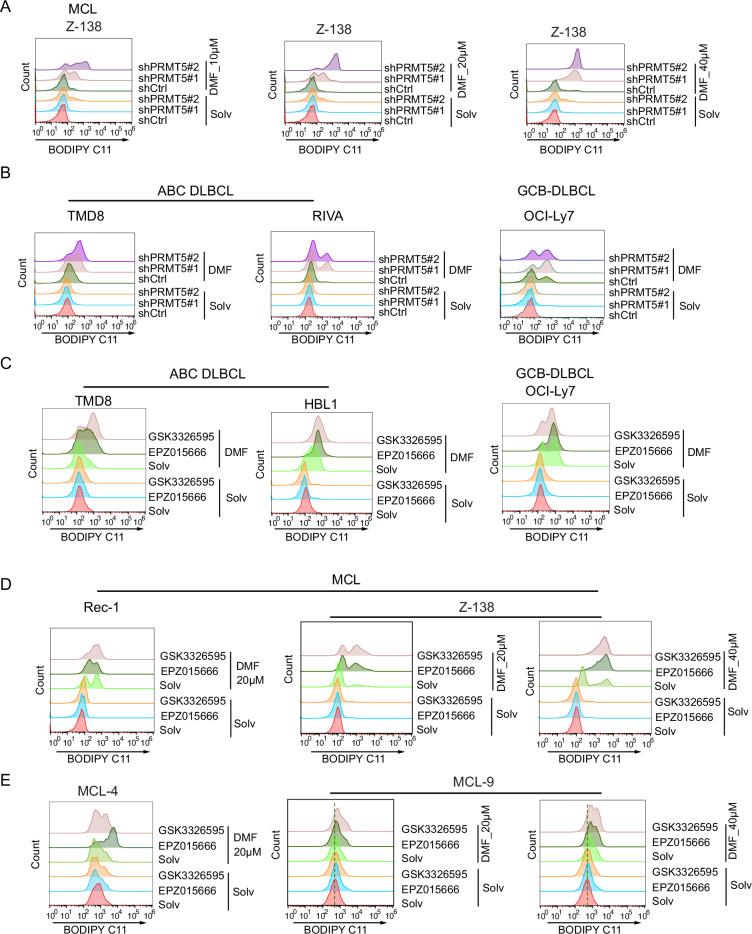
PRMT5 inhibition promotes DMF-induced lipid peroxidation in DLBCL and MCL cells. **A** Z-138 cells transduced with two independent PRMT5-targeting shRNAs or a control shRNA were treated with dimethyl fumarate (DMF) at 10, 20, or 40 µM for 3 h. Lipid peroxidation was assessed by flow cytometry using the oxidation-sensitive fluorescent probe BODIPY C11. **B** Flow cytometric analysis of lipid peroxidation in DLBCL cell lines after 3 h of treatment with 20 µM DMF in indicated cells. **C** Flow cytometric analysis of lipid peroxidation in DLBCL cell lines treated with the PRMT5 inhibitors GSK3326595 (1 µM) or EPZ015666 (1 µM), or vehicle control, for 5 days, followed by 3 h of treatment with 20 µM DMF. **D** Flow cytometric analysis of lipid peroxidation in MCL cell lines treated with GSK3326595 (1 µM) or EPZ015666 (1 µM), or vehicle control, for 5 days, followed by DMF treatment at the indicated concentrations for 3 h. **E** Flow cytometric analysis of lipid peroxidation in MCL cell lines and primary MCL cells treated with GSK3326595 (1 µM) or EPZ015666 (1 µM), or vehicle control, for 5 days, followed by treatment with DMF at the indicated concentrations for 3 h.

We reanalyzed RNA-seq data from our recent study on two representative DLBCL cell lines, OCI-Ly7 and TMD8 [28]. The results revealed that common gene signatures enriched in both cell lines upon PRMT5 knockout include PI3K-AKT-mTOR, MYC targets, and E2F targets, as expected. Interestingly, PRMT5 also regulates various aspects of cellular metabolism, including fatty acid metabolism, cholesterol homeostasis, glycolysis and lipid peroxidation (Supplemental Fig. 1A, B), suggesting that PRMT5 may be involved in ferroptosis. To test whether PRMT5 regulates ferroptosis, we quantified lipid peroxidation in MCL and DLBCL cells using the oxidation-sensitive lipophilic probe BODIPY C11 after PRMT5 knockdown or inhibition, in the presence of DMF or its solvent control. Flow cytometric analysis demonstrated that PRMT5 knockdown did not induce apparent lipid peroxidation but significantly enhanced DMF-mediated ferroptosis in all tested cell lines, including one MCL cell line, two ABC DLBCL cell lines, and one GCB DLBCL cell line (Fig. 1A, B). This effect was dose-dependent (Fig. 1A). A similar result was observed when PRMT5 was inhibited with two specific inhibitors (Fig. 1C, D). Notably, the two PRMT5 inhibitors also enhanced DMF-mediated lipid peroxidation in two MCL patient samples (MCL-4 and MCL9) (Fig. 1E). The knockdown efficiency of both PRMT5 shRNAs in these cell lines was confirmed by immunoblotting (Supplemental Fig. 2A). We also confirmed the efficacy of both PRMT5 inhibitors by assessing symmetric dimethylation of the target protein H4R3 in these cell lines and patient samples (supplemental Fig. 2B). Of note, a 3 h DMF treatment following with 5-day pretreatment with the same concentrations of the two PRMT5 inhibitors, as used in the ferroptosis assays above, did not induce cell apoptosis (supplemental Fig. 3A–D). Together, these data suggest that PRMT5 plays a role in the regulation of lipid peroxidation and ferroptosis.

### PRMT5 inhibition suppresses SLC7A11 expression in DLBCL and MCL cells

**Fig. 2:**
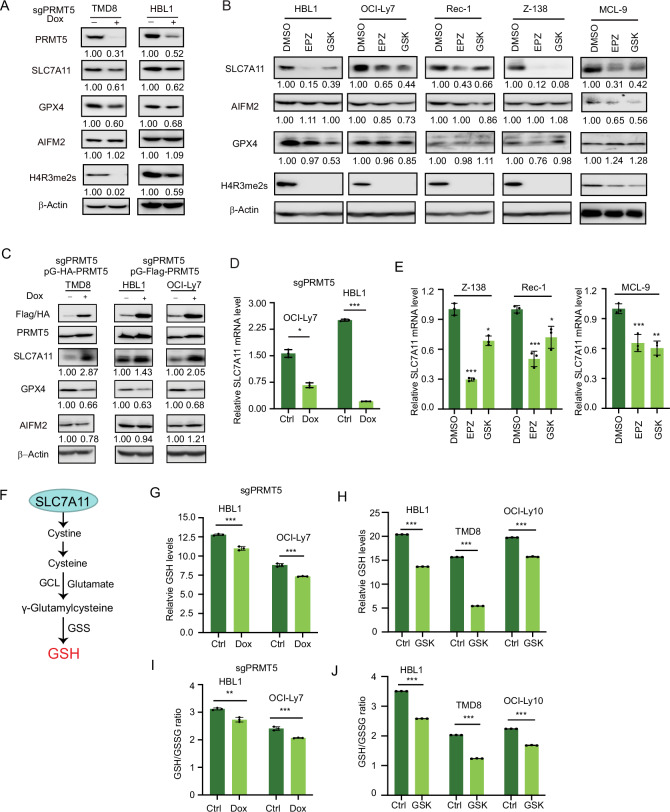
PRMT5 inhibition suppresses SLC7A11 expression in DLBCL and MCL cells. **A** Immunoblot analysis of SLC7A11, GPX4, and AIFM2 proteins in TMD8 and HBL1 cells following PRMT5 knockout using sgRNA. PRMT5 sgRNA expression was induced with 20 ng/mL doxycycline for 6 days prior to analysis. Note the reduction in H4R3 symmetric dimethylation (H4R3me2s) following PRMT5 knockout. β-Actin was used as a loading control. **B** Immunoblot analysis of SLC7A11, GPX4, and AIFM2 proteins in DLBCL, MCL cell lines, and primary MCL cells treated with the PRMT5 inhibitors GSK3326595 (1 µM) or EPZ015666 (1 µM) for 5 days. **C** Immunoblot analysis of SLC7A11, GPX4, and AIFM2 proteins in DLBCL cells following exogenous overexpression of HA-tagged or FLAG-tagged PRMT5 in PRMT5 knockout backgrounds. The overexpressed PRMT5 constructs contained mutations at the sgRNA target sites to prevent re-editing. PRMT5 overexpression was induced with 20 ng/mL doxycycline for 5 days prior to analysis. **D**, **E** Quantitative real-time PCR analysis of SLC7A11 mRNA expression relative to β-actin in OCI-Ly7 and HBL1 cells (D), and in MCL cells (**E**). **F** Schematic model of the glutathione (GSH) synthesis pathway. Glutamate and cysteine are first conjugated by glutamate–cysteine ligase (GCL) to form γ-glutamylcysteine. Subsequently, glycine is added by glutathione synthetase (GSS) to generate GSH. This pathway highlights the sequential enzymatic steps essential for intracellular GSH biosynthesis. **G** Reduced GSH levels following PRMT5 knockout. PRMT5 sgRNA expression was induced with 20 ng/mL doxycycline for 6 days prior to analysis. **H** Reduced GSH levels after 5 days of treatment with 1 µM GSK3326595. **I** Reduced ratios of glutathione (GSH) to oxidized glutathione (GSSG) after 6 days of PRMT5 sgRNA expression. **J** Reduced ratios of GSH to GSSG after 5 days of treatment with 1 µM GSK3326595. Data in all panels represent mean ± SD of three biological replicates (*p  <  0.05, **p  <  0.01, ***p  <  0.001; *n*  =  3).

To investigate the role of PRMT5 in regulating ferroptosis, we first conducted immunoblotting to assess the expression of three major ferroptosis-inhibitory proteins, SLC7A11, GPX4, and AIFM2, following PRMT5 inhibition. The results showed a reduction in SLC7A11 and GPX4 protein levels, but not AIFM2, in two cell lines following PRMT5 knockout (Fig. 2A). Similarly, treatment with two PRMT5 inhibitors led to a significant decrease in SLC7A11 expression in two DLBCL cell lines, two MCL cell lines, and one MCL patient sample, whereas GPX4 expression was either unchanged or only slightly reduced (Fig. 2B). Consistent with these findings, AIFM2 expression remained largely unchanged, with the exception of a moderate decrease observed in primary MCL-9 cells (Fig. 2B). Next, we performed a rescue experiment in which PRMT5 cDNA, containing mutations in the sgRNA target sequence, was retrovirally expressed in cells with endogenous PRMT5 knockout. The results showed that PRMT5 overexpression significantly increased SLC7A11 expression, but not GPX4 or AIFM2 (Fig. 2C). Further quantitative PCR analysis confirmed a reduction in SLC7A11 mRNA following PRMT5 knockout in DLBCL cells (Fig. 2D) or PRMT5 inhibition in two MCL cell lines and one MCL patient sample (Fig. 2E). We did not detect a physical interaction between PRMT5 and SLC7A11 (supplemental Fig. 2C). Of note, 20 ng/ml doxycycline used for sgRNA expression did not affect the expression of SLC7A11, GPX4, or AIFM2 (supplemental Fig. 2D). These findings suggest that PRMT5 regulates SLC7A11 at the transcriptional level rather than through posttranslational modification. A recent study demonstrated that PRMT5 suppresses ferroptosis by methylating GPX4, which stabilizes the GPX4 protein [45]. However, our data suggest that PRMT5 has a limited effect on this mechanism in DLBCL and MCL cells, indicating that its role in regulating GPX4 may be cell type-specific.

As shown in Fig. 2F, SLC7A11 imports cystine into the cell, where it is rapidly reduced to cysteine. Intracellular cysteine serves as a critical antioxidant and is the rate-limiting substrate for glutamate–cysteine ligase (GCL), which catalyzes the first step in GSH synthesis [37]. The second step is catalyzed by glutathione synthetase (GSS), which completes the formation of GSH [37]. To assess the functional impact of PRMT5 on the regulation of SLC7A11, we measured intracellular GSH levels and observed a reduction in GSH production following PRMT5 inhibition, either by sgRNA (Fig. 2G) or a specific inhibitor (Fig. 2H), in these cell lines. Consistently, the ratio of reduced GSH to its oxidized form (GSSG) also decreased upon PRMT5 inhibition, whether by sgRNA (Fig. 2I) or the inhibitor (Fig. 2J). Taken together, these results suggest that PRMT5 inhibits ferroptosis by upregulating SLC7A11, which increases the import of cystine into the cell for GSH synthesis.

### PRMT5 upregulates ATF5, leading to increased expression of SLC7A11 and ATF4 in DLBCL and MCL cells

Reanalysis of RNA-seq data from our recent study [28] revealed that the transcription factor ATF5 is the most significantly regulated and abundantly expressed gene after PRMT5 knockout (supplemental Fig. 4A). To extend these findings, we reanalyzed RNA-seq data (GSE125966) from the GOYA trial cohort, which includes 553 de novo DLBCL patients [46]. Of note, high expression levels of ATF5, but not ATF4, are associated with poor overall survival in DLBCL (Supplemental Fig. 4B, C). Interestingly, higher expression levels of both ATF5 and ATF4 correlate with worse survival compared to lower expression levels (Supplemental Fig. 4D). A similar trend was observed in MCL, based on our reanalysis of Affymetrix gene expression data from 123 MCL samples in a study [47] (supplemental Fig. 4E–G). Previous studies have shown that ATF5 deficiency reduces antioxidant proteins and increases mitochondrial ROS production [48], while its close family member ATF4 suppresses ferroptosis by regulating SLC7A11 expression [49, 50]. These findings prompted us to investigate a potential role for ATF5 in the regulation of ferroptosis.

**Fig. 3:**
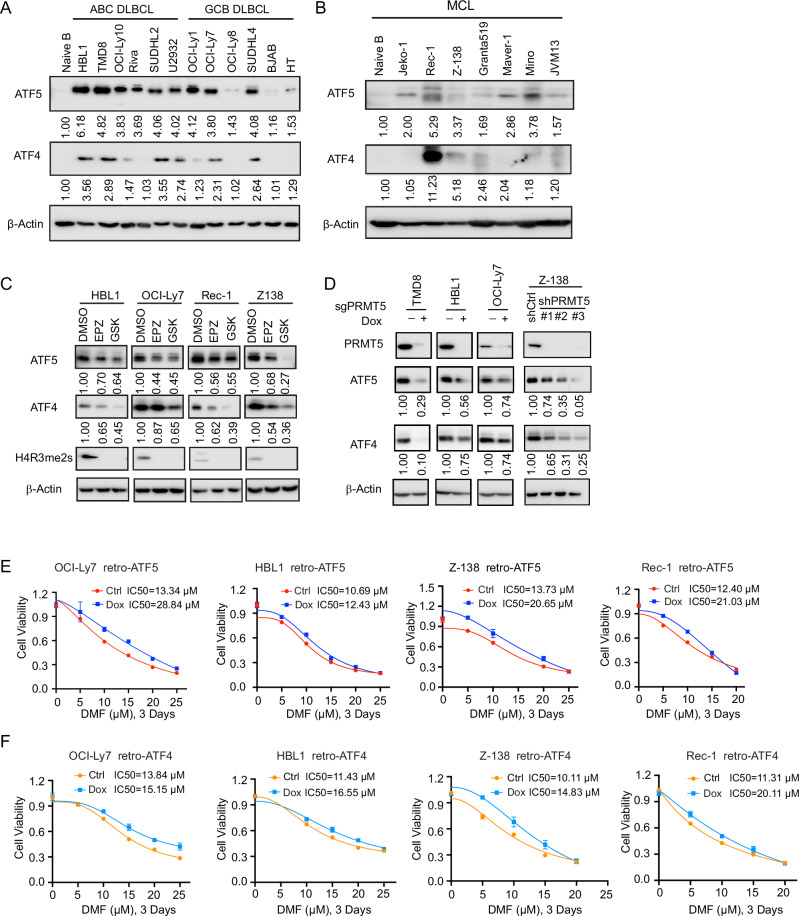
PRMT5 inhibition reduces the expression of ATF5 and ATF4 in DLBCL and MCL. **A**, **B** Immunoblot analysis of ATF5 and ATF4 expression in naive B cells and various DLBCL cell lines (**A**) and MCL cell lines (**B**). β-Actin was used as a loading control. **C** Immunoblot analysis of ATF5, ATF4, and H4R3me2s expression in DLBCL (HBL1, OCI-Ly7) and MCL (Rec-1, Z-138) cell lines treated with the PRMT5 inhibitors EPZ015666 (1 µM) and GSK3326595 (1 µM) for 5 days. **D** Immunoblot analysis of PRMT5, ATF5, and ATF4 expression in PRMT5 sgRNA-induced knockout DLBCL cell lines (TMD8, HBL1, OCI-Ly7) and PRMT5 shRNA constitutive knockdown MCL cell line (Z-138). Expression of PRMT5 sgRNA was induced with 20 ng/mL doxycycline for 6 days. **E**,**F** Inducible overexpression of ATF5 (**E**) or ATF4 (**F**) using the retro-CMV-TO-PG-ATF5 or ATF4 vector in DLBCL and MCL cells increased resistance to DMF treatment, as measured by the CellTiter-Glo™ Luminescent Cell Viability Assay. ATF5 or ATF4 expression was induced with 20 ng/mL doxycycline for 2 days, followed by 3 days of DMF treatment. IC₅₀ values were calculated using GraphPad Prism (v9.0) with a four-parameter nonlinear regression model.

We first screened DLBCL and MCL cell lines for ATF4 and ATF5 expression using immunoblotting and found that their expression was increased in all of these cell lines compared to normal naive B cells, although the levels varied between cell lines (Fig. 3A, B). Of note, unlike ATF5, ATF4 expression is very low and barely detectable in some cell lines, similar to that in naive B cells (Fig. 3A, B). Further immunoblotting analysis demonstrated reduced expression of both ATF5 and ATF4 following PRMT5 inhibition, either by two specific inhibitors (Fig. 3C) or by its sgRNA or its shRNAs (Fig. 3D). Functional analysis showed reduced sensitivity of DLBCL and MCL cells to DMF following overexpression of ATF5 (Fig. 3E) or ATF4 (Fig. 3F). This suggests that, like ATF4, ATF5 is involved in the regulation of ferroptosis.

**Fig. 4:**
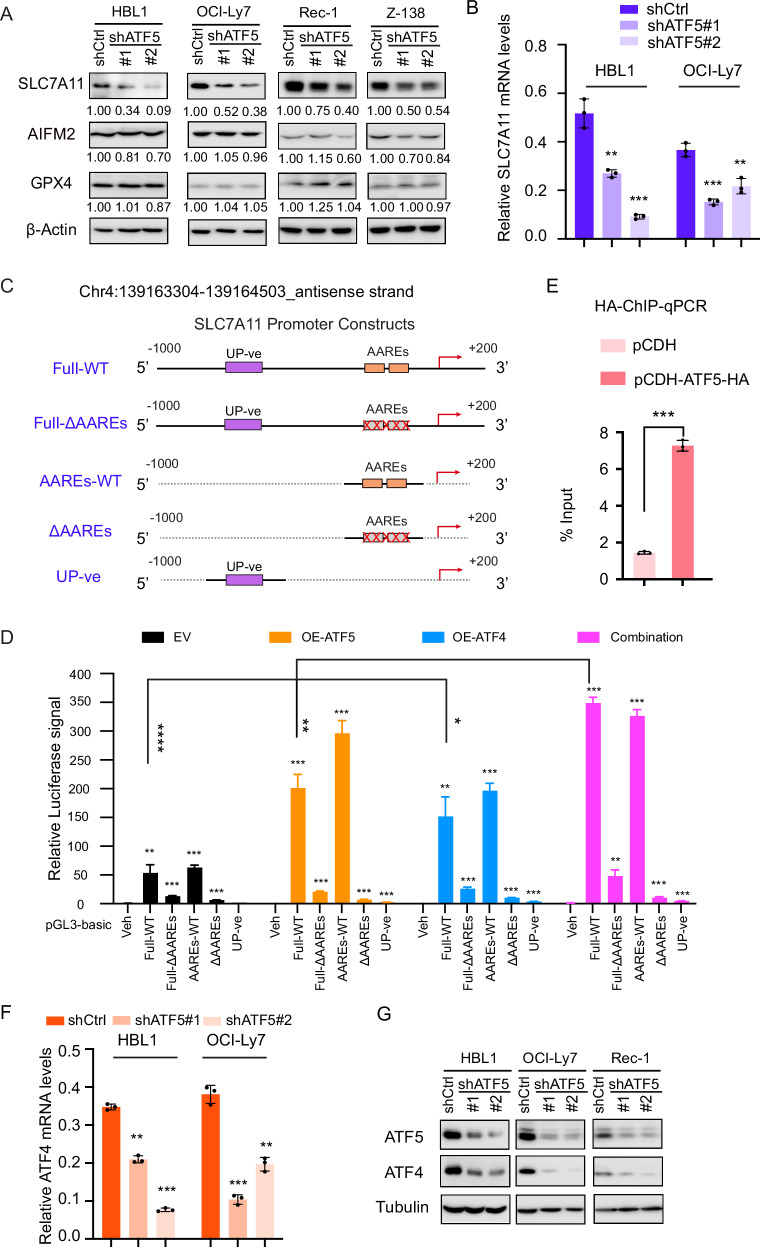
ATF5 induces SLC7A11 expression in DLBCL and MCL. **A** Immunoblot analysis of AIFM2, GPX4, and SLC7A11 protein levels following ATF5 knockdown using two independent shRNAs or a control shRNA in HBL1, OCI-Ly7, Rec-1 and Z138 cells. β-Actin was used as a loading control. ATF5 knockdown was induced with 20 ng/mL doxycycline for 4 days. **B** Quantitative real-time PCR analysis of SLC7A11 mRNA expression relative to β-actin in OCI-Ly7 and HBL1 cells following ATF5 knockdown. Data represent the mean ± SD from three biological replicates (**p  <  0.01, ***p  <  0.001; *n * =  3). **C** Schematic representation of SLC7A11 promoter constructs used in luciferase reporter assays. Full-WT includes a 1200-bp region spanning –1000 to +200 bp relative to the transcription start site (TSS), containing two AARE elements. Full-ΔAAREs denotes the same promoter region with both AARE elements deleted. AAREs-WT represents a 260 bp fragment containing AARE1 and AARE2. ΔAAREs refers to the AAREs-WT fragment with both AARE elements removed. UP-ve represents a 260 bp upstream fragment of the SLC7A11 promoter lacking AAREs. **D** Dual-luciferase reporter assay in 293 T cells demonstrating that ATF5 and/or ATF4 bind to the SLC7A11 promoter and activate its transcription. Firefly luciferase activity from the pGL3-basic reporter was normalized to β-galactosidase activity. Data represent the mean ± SD from three independent experiments (*p  <  0.05, **p  <  0.01, ***p  <  0.001, ****p < 0.0001; *n*  =  3). **E** HA ChIP-qPCR analysis was performed in 293 T cells transfected with either empty vector (pCDH) or pCDH-ATF5-HA. Primers targeting the promoter and transcription start site (TSS) regions of the SLC7A11 gene were used to assess ATF5 binding. Data represent the mean ± SD from three independent experiments (***p  <  0.001; *n*  =  3). **F** Quantitative real-time PCR analysis showing reduced mRNA expression of ATF4 relative to β-actin in HBL1 and OCI-Ly7 cells following doxycycline (Dox)-inducible ATF5 shRNA knockdown (20 ng/mL for 4 days). Data represent mean ± SD from three biological replicates (**p  <  0.01, ***p  <  0.001; *n*  =  3). **G** Immunoblot analysis demonstrating decreased ATF5 and ATF4 protein levels in HBL1, OCI-Ly7, and Rec-1 cells following ATF5 knockdown using two independent Dox-inducible shRNAs (20 ng/mL Dox for 4 days).

We next tested whether PRMT5-mediated SLC7A11 expression is regulated through ATF5. Immunoblotting demonstrated reduced SLC7A11 protein expression, but not GPX4 or AIFM2, following ATF5 knockdown with two independent shRNAs (Fig. 4A). Knockdown of ATF5 also reduced the mRNA levels of SLC7A11 (Fig. 4B), suggesting that ATF5 functions as a transcription factor for SLC7A11. It is known that the homodimers or heterodimers of ATF4 and ATF5 mediate their transcriptional function [51]. To functionally test ATF5-mediated regulation of SLC7A11, we performed a standard dual-luciferase reporter assay in 293 T cells. In addition to the full-length promoter of SLC7A11, we generated a construct containing the AARE1 and AARE2 regions, which are known binding sites for ATF5 and ATF4 [50, 52]. We also included two deletion constructs lacking the AARE1 and AARE2 elements, as well as an upstream negative control (Fig. 4C). The results showed that both ATF5 and ATF4 exhibited transcriptional activity when the reporter constructs contained the full-length promoter or the AARE1/AARE2 regions, but not when the AARE1/2 regions were deleted or when the negative control was used (Fig. 4D). The binding of ATF5 to the SLC7A11 promoter regions was further confirmed by ATF5-HA ChIP-qPCR analysis (Fig. 4E). Interestingly, the combined expression of ATF5 and ATF4 slightly increased transcriptional activity compared to their individual expression, suggesting that ATF5 may dimerize with ATF4. To test this possibility, we performed co-immunoprecipitation (co-IP) in 293 T cells. The results revealed a physical interaction between the two proteins (Supplemental Fig. 5A, B).

**Fig. 5:**
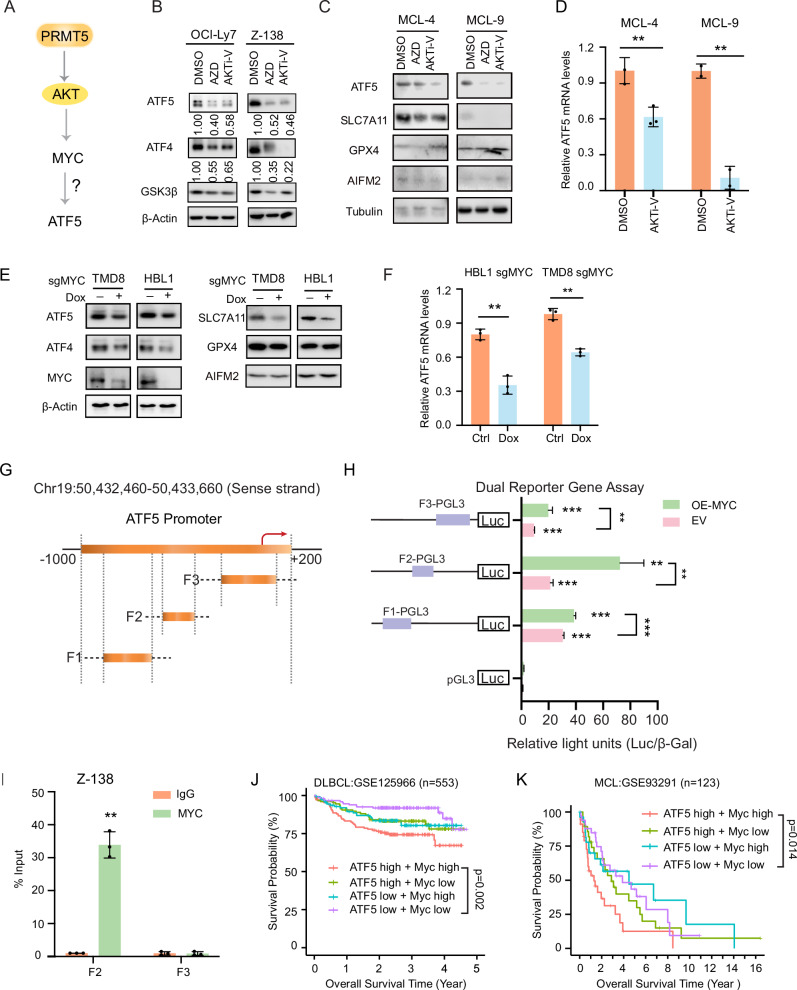
MYC induces ATF5 expression in DLBCL and MCL cells. **A** Schematic model illustrating the PRMT5–AKT–MYC–ATF5 signaling axis. PRMT5 modulates AKT activation, leading to MYC stabilization and subsequent transcriptional upregulation of ATF5, contributing to downstream gene expression changes. **B** Immunoblot analysis of ATF5, ATF4, and GSK3β protein levels following treatment with AKT inhibitors (1 µM AZD5356 and 1 µM AKTi-V) for 3 days in OCI-Ly7 and Z-138 cells. β-Actin was used as a loading control. **C** Immunoblot analysis of ATF5, SLC7A11, GPX4 and AIFM2 protein levels in MCL-4 and MCL-9 primary cells following treatment with AKT inhibitors (1 µM AZD5356 and 1 µM AKTi-V) for 5 days. Tubulin was used as a loading control. **D** Quantitative real-time PCR analysis of ATF5 mRNA expression relative to β-actin in MCL-4 and MCL-9 cells after 1 µM AKTi-V treatment. Data represent mean ± SD from three biological replicates (**p  <  0.01; *n*  =  3). **E** Immunoblot analysis of ATF5, ATF4, SLC7A11, GPX4, AIFM2, and MYC protein levels following MYC knockout using sgRNA in TMD8 and HBL1 cells. β-Actin was used as a loading control. MYC sgRNA expression was induced with 20 ng/mL doxycycline for 5 days prior to analysis. **F** Quantitative real-time PCR analysis of ATF5 mRNA expression relative to β-actin in HBL1 and TMD8 cells following MYC knockout. MYC sgRNA expression was induced with 20 ng/mL doxycycline for 5 days. Data represent the mean ± SD from three biological replicates (**p  <  0.01; *n*  =  3). **G** Schematic representation of truncated ATF5 promoter constructs used in luciferase reporter assays. **H** Dual-luciferase reporter assay in 293 T cells demonstrating that MYC binds to the ATF5 promoter and activates its transcription. Firefly luciferase activity from the pGL3-basic reporter was normalized to β-galactosidase activity. Data represent the mean ± SD from three independent experiments (**p  <  0.01, ***p  <  0.001; *n*  =  3). **I** MYC ChIP-qPCR analysis using two primer pairs targeting the ATF5 promoter and transcription start site regions. Immunoglobulin G (IgG) served as a negative control. Error bars represent the mean ± SD (**p  <  0.01; *n * =  3). **J**, **K** Kaplan–Meier survival curves of DLBCL (J; *n* = 553, GSE125966) and MCL (K; *n* = 123, GSE93291) patients stratified by combined ATF5 and MYC expression levels. Patients were grouped into four categories based on whether each gene’s expression was above or below the median (“high” or “low”). Survival differences were assessed using the log-rank (Mantel–Cox) test; overall p-values are shown. Pairwise comparisons were performed using pairwise log-rank tests. Confidence intervals are not shown.

ATF4 is a known target gene of MYC [53, 54] and inhibits ferroptosis by upregulating SLC7A11 [49, 50]. We found that ATF5 also regulates ATF4 expression. Knockdown of ATF5 significantly reduced both ATF4 mRNA and protein levels in DLBCL and MCL cells (Fig. 5F, G and supplemental Fig. 6A). Conversely, AFT5 overexpression increased ATF4 expression (supplemental Fig. 6B). ATF5-mediated transcription of ATF4 was further supported by a dual-luciferase reporter assay, in which MYC served as a positive control (supplemental Fig. 6C–E). In sum, these data suggest that ATF5 is a critical transcription factor in the biology of DLBCL and MCL, promoting the expression of SLC7A11 and ATF4, two key genes involved in the inhibition of ferroptosis.

### ATF5 expression is induced through the PRMT5-AKT-MYC axis

We reanalyzed RNA-seq data [28] and did not observe changes in the splicing patterns of ATF4 and ATF5 following PRMT5 knockout (supplemental Fig. 7A, B). Therefore, ATF5 regulation by PRMT5 is unlikely to be due to splicing. Co-immunoprecipitation analysis did not support a physical interaction between PRMT5 and ATF5 (supplemental Fig. 7C), suggesting that ATF5 is not a direct methylation target of PRMT5. We next tested whether PRMT5-mediated ATF5 expression is regulated through the AKT-MYC axis. As illustrated in Fig. 5A, PRMT5 mediates PI3K-AKT-MYC signaling to promote the proliferation and survival of both ABC and GCB DLBCL cells [28]. It is possible that PRMT5 regulates ATF5 expression through this pathway, where ATF5 could be a target of MYC, a significant oncoprotein in various lymphomas, including DLBCL and MCL [44, 55, 56]. To test this, we first performed ATK inhibition experiments and found that the expression ATF5 and ATF4 was reduced following treatment with two AKT inhibitors, AZD5356 and AKTi-V, in both DLBCL and MCL cells (Fig. 5B). As expected, these ATK inhibitors reduced levels of GSK3β (Fig. 5B), a direct target of AKT [57]. Reduced ATF5 expression was also observed in two MCL patient samples following AKT inhibitor treatment (Fig. 5C, D). We then knocked out MYC in two cell lines and observed a reduction in the expression of ATF5, ATF4, and SLC7A11, but not GPX4 or AIFM2 (Fig. 5E). MYC knockout also reduced ATF5 mRNA levels (Fig. 5F), suggesting that ATF5 is a direct target of MYC.

We then tested MYC transcriptional activity on ATF5 using the dual-luciferase reporter assay described above. Since there are no typical E-box regions in the ATF5 promoter, we searched the ENCODE MYC ChIP-seq data for hepatocellular carcinoma HepG2 cells and found enrichment of MYC binding sites in the F1 and F2 regions (Fig. 5G). The results demonstrated a significantly increased signal in the F2 region, but less so in the F1 and transcription start site (F3) regions (Fig. 5H). These findings were confirmed by MYC ChIP-qPCR analysis in MCL cells (Fig. 5I). Interestingly, after reanalyzing gene expression data from two datasets, we found that higher expression levels of both ATF5 and MYC correlate with worse survival compared to lower expression levels in patients with DLBCL (Fig. 5J) and MCL (Fig. 5K) following immunochemotherapy. Therefore, these findings suggest that ATF5 is regulated by the PRMT5-AKT-MYC axis, and that the combined expression of MYC and ATF5 may serve as a prognostic biomarker for both DLBCL and MCL.

### PRMT5 inhibition sensitizes DLBCL and MCL cells to ferroptosis

**Fig. 6:**
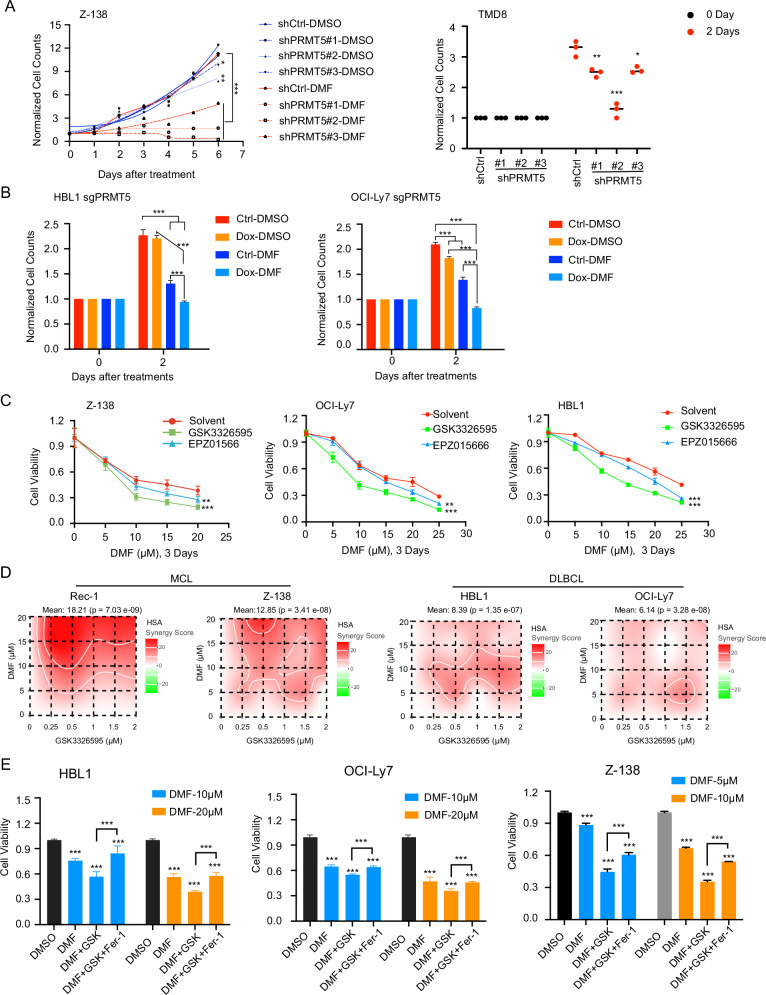
PRMT5 inhibition sensitizes DLBCL and MCL cells to DMF treatment. **A** Left panel**:** Knockdown of PRMT5 using three independent shRNAs or a control vector in Z-138 cells sensitized the cells to DMF treatment. Fresh 20 µM DMF was added on Day 0 and Day 3, and cell numbers were assessed by trypan blue exclusion assay at the indicated time points. Error bars represent the mean ± SD from three biological replicates (*p  <  0.05, **p  <  0.01, ***p  <  0.001). Right panel: knockdown of PRMT5 using three independent shRNAs or a control vector in TMD8 cells sensitized the cells to DMF treatment. Cell numbers were assessed by trypan blue exclusion assay at Day 0 and Day 2. Error bars represent the mean ± SD from three biological replicates (*p  <  0.05, **p  <  0.01, ***p  <  0.001). **B** Knockout of PRMT5 using sgRNA in HBL1 (left panel) and OCI-Ly7 (right panel) cells sensitized the cells to DMF treatment. Cell numbers were assessed by trypan blue exclusion assay at Day 0 and Day 2. PRMT5 knockout was induced with 20 ng/mL doxycycline for 5 days, followed by 3 days of 20 µM DMF treatment. Error bars represent the mean ± SD from three biological replicates (***p  <  0.001). **C** PRMT5 inhibition using 1 µM EPZ015666 or 1 µM GSK3326595 for 5 days sensitized Z-138, OCI-Ly7, and HBL1 cells to DMF treatment, as measured by the CellTiter-Glo™ Luminescent Cell Viability Assay. Error bars represent the mean ± SD from three biological replicates (**p  <  0.01, ***p  <  0.001). **D** Synergy analysis of the PRMT5 inhibitor GSK3326595 in combination with DMF in the indicated MCL and DLBCL cell lines for 3 days. Synergy scores were calculated using SynergyFinder 2.0. A synergy score > 0 (pink) indicates a synergistic interaction between the two drugs. **E** The ferroptosis inhibitor ferrostatin-1 (Fer-1) rescued the growth inhibition caused by the combination of GSK3326595 and DMF in HBL1, OCI-Ly7, and Z-138 cells for 3 days, as measured by the CellTiter-Glo™ Luminescent Cell Viability Assay. Error bars represent the mean ± SD from three biological replicates (***p  <  0.001).

To determine whether PRMT5 inhibition sensitizes DLBCL and MCL cells to ferroptotic cell death, we assessed cell viability using both genetic and pharmacological approaches to inhibit PRMT5 in combination with DMF treatment. The results showed a significant reduction in cell viability when PRMT5 was inhibited using either shRNAs (Fig. 6A) or a CRISPR/Cas9-mediated sgRNA (Fig. 6B), compared to treatment with either agent alone. Similarly, enhanced DMF-induced ferroptotic cell death was observed when cells were treated with two different PRMT5 inhibitors (Fig. 6C), and the combination exhibited a synergistic effect (Fig. 6D). Synergy was also seen between GSK3326595 and two ferroptosis inducers, the GPX4 inhibitor RSL3 (supplemental Fig. 8A) and the SLC7A11 inhibitor Erastin (supplemental Fig. 8B). This confirmed proof of principle for the anti-tumor synergy between PRMT5 inhibitors and ferroptosis inducers. Of note, the reduced cell viability observed with co-treatment of the PRMT5 inhibitor GSK3326595 and DMF was rescued by the ferroptosis inhibitor Ferrostatin-1 (Fer-1) (Fig. 6E). In addition, N-acetylcysteine (NAC), a precursor of GSH, exhibited a similar rescue effect, whereas the pan-caspase inhibitor quinolyl-valyl-O-methylaspartyl-[2,6-difluorophenoxy]-methyl ketone (Q-VD-OPh) did not (supplemental Fig. 8C–E). Together, these data suggest that the cell death is ferroptosis-dependent.

**Fig. 7:**
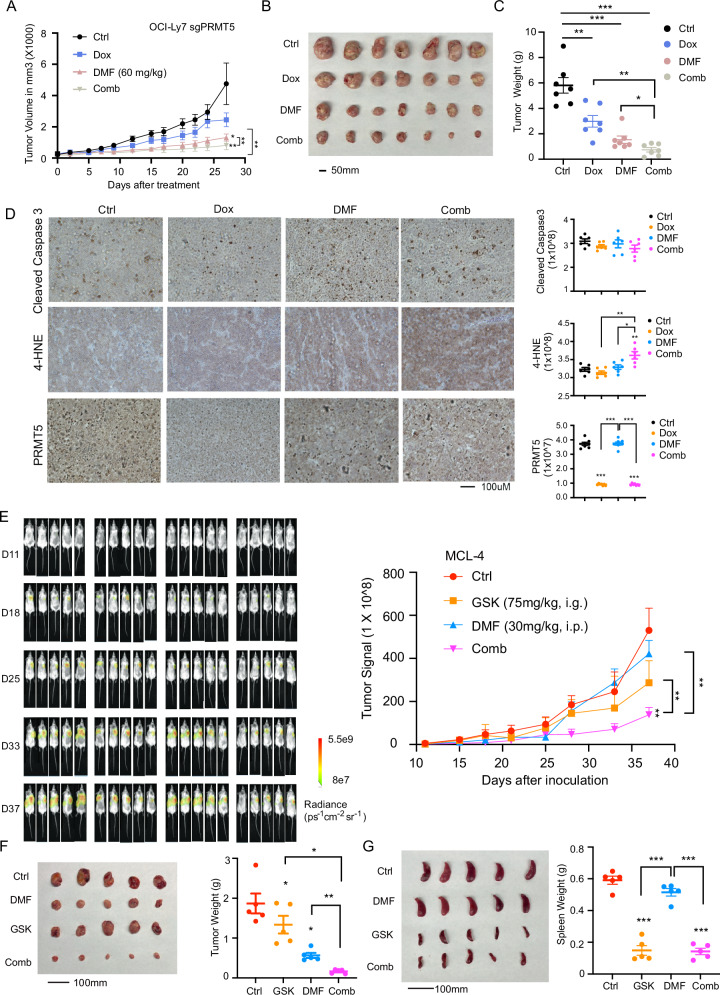
PRMT5 inhibition enhances the anti-tumor effects of DMF. **A** DLBCL OCI-Ly7 PRMT5 sgRNA xenograft model. OCI-Ly7 cells expressing PRMT5-targeting sgRNA were implanted subcutaneously into NSG mice. Once tumors reached an average volume of 100 mm³, mice were treated with 60 mg/kg DMF (intraperitoneally, daily), 2 mg/mL doxycycline in drinking water, or a combination of both until the study endpoint (Day 27). Tumor growth was monitored throughout the treatment period. Error bars represent the mean ± SEM (two-way ANOVA; *p  <  0.05, **p  <  0.01, *n* = 7)). **B** Representative images of tumors from each treatment group. Scale bar, 50 μm. Comb, combination treatment. **C** Tumor weights from each group at the study endpoint. Error bars represent the mean ± SEM (*p < 0.05, **p < 0.01, ***p  <  0.001, *n* = 7). **D** Representative immunohistochemical staining of cleaved caspase-3, 4-HNE and PRMT5 in tumor tissues from each treatment group (left panel, scale bar, 100 μm). “Comb” denotes the combination treatment. Quantification of protein expression levels were performed using Fiji (ImageJ) software (right panel). **E** PDX model of MCL. Freshly isolated MCL-4 cells were inoculated subcutaneously into NSG mice. Tumor burden was monitored using the Lago X bioluminescence imaging system (Spectral Instruments Imaging). On Day 10 post-inoculation, mice were randomized into four treatment groups (*n* = 5). Beginning on Day 11, mice were treated with 75 mg/kg GSK3326595 (orally, every other day), 30 mg/kg DMF (intraperitoneally, every other day), or a combination of both drugs for 4 weeks. For combination treatment, GSK3326595 and DMF were administered on alternating days to avoid simultaneous dosing. Representative bioluminescence images (left) and tumor growth curves (right) are shown. The color scale indicates photon flux (photons/second) emitted by tumors. Error bars represent the mean ± SEM (two-way ANOVA; **p  <  0.01). **F** Photographs of tumors (left) and quantification of tumor weights (right) from each treatment group (*n* = 5). Error bars represent the mean ± SEM (*p  <  0.05, **p  <  0.01). **G** Photographs of spleens (left) and spleen weights (right) from each treatment group. Error bars represent the mean ± SEM (***p  <  0.001, *n* = 5).

To confirm this finding in vivo, we conducted cell line-derived xenograft (CDX) and patient-derived xenograft (PDX) experiments using immunocompromised NOD scid γc^-/-^ (NSG) mice. In the CDX model, PRMT5 knockout via sgRNA alone resulted in a modest reduction in tumor growth, but significantly enhanced the DMF-mediated suppression of tumor progression, as evidenced by reductions in tumor volume (Fig. 7A), size (Fig. 7B), and weight (Fig. 7C). There were no changes in body weight in recipient mice from any group (supplemental Fig. 10A). Of note, the combination of PRMT5 knockout and DMF significantly increased the production of 4-hydroxynonenal (4-HNE), a key lipid peroxidation product [58], without altering levels of cleaved caspase-3 (Fig. 7D), suggesting that ferroptosis contributes to tumor growth inhibition.

We next performed PDX experiments using MCL-4 cells from a MCL patient who had relapsed following immunochemotherapy, as described in our recent studies [43, 44]. To expand the tumor cells, 2 million cells were injected intravenously into sublethally irradiated (2 Gy) NSG mice (supplemental Fig. 10B). Thirty-five days post-injection, flow cytometric analysis of cells isolated from liver and spleen tissues revealed that 85–90% of the cells were tumor cells. These cells maintained their phenotypic identity, as indicated by persistent expression of human CD19 and CD20 (B cell markers), and lack of expression of human CD38 (a marker of B cell differentiation) (supplemental Fig. 9C–E). Ten days after tumor inoculation using cells isolated from the liver of a donor mouse, we initiated treatment in NSG mice with either 75 mg/kg GSK3326595 (administered orally every other day), 30 mg/kg DMF (administered intraperitoneally every other day), or a combination of both drugs for a duration of 4 weeks (supplemental Fig. 10B). The results showed that tumor growth was significantly reduced in the combination treatment group compared to either single-agent or vehicle control groups (Fig. 7E–G), indicating that PRMT5 inhibition enhances the sensitivity of MCL-4 cells to DMF. Importantly, no overt toxicities were observed in the treated MCL PDX mice, and body weights remained stable across all treatment groups (supplemental Fig. 10C). A similar reduction in tumor growth was observed in MCL-4 cells isolated from the spleen of a donor mouse (supplemental Fig. 10D–F). Taken together, these findings suggest that combining a PRMT5 inhibitor with DMF may represent a promising therapeutic strategy for patients with DLBCL or MCL who are refractory to or have relapsed following immunochemotherapy.

## Discussion

In this study, we reveal a role for PRMT5 in regulating ferroptosis in DLBCL and MCL cells. PRMT5 suppresses ferroptosis through upregulation of SLC7A11, which is mediated by the AKT-MYC-ATF5 axis. In addition to inducing SLC7A11 expression, ATF5 is overexpressed in these lymphoma cells and functions as a transcription factor to promote the expression of ATF4, a known ATF family member involved in regulating ferroptosis. We demonstrate that either genetic or pharmacological inhibition of PRMT5 sensitizes DLBCL and MCL cells to ferroptosis inducers, including DMF. Use of the PRMT5 inhibitor GSK3326595 enhances the anti-tumor effects of DMF in a PDX animal model.

The PRMT5-AKT-MYC-ATF5-SLC7A11 axis is essential for the fitness of DLBCL and MCL cells. Knockout of PRMT5 or MYC increases DMF-mediated ferroptotic cell death and reduces cell viability in these lymphoma cells (supplemental Fig. 11A, B). However, this effect can be rescued by overexpression of SLC7A11 (supplemental Fig. 11A, B), suggesting that defense against ferroptosis is critical for cell survival. A recent study demonstrated that PRMT5 inhibits ferroptosis by methylating GPX4, thereby preventing GPX4 from binding to the Cullin1-FBW7 E3 ligase and thus blocking ubiquitination-mediated GPX4 degradation [45]. Interestingly, our data suggest that PRMT5 has a limited effect on GPX4 regulation in lymphoma cells. It would be interesting to further dissect the role of GPX4 in the biology of B-cell lymphomas, including DLBCL and MCL.

Currently, all ferroptosis inducers, including the GPX4 inhibitor RSL3 and the SLC7A11 inhibitor Erastin, are restricted to preclinical studies and have not advanced to clinical trials due to their general cytotoxicity [37, 40, 59]. FDA-approved DMF offers a unique opportunity to target ferroptosis in the clinic. DMF triggers ferroptotic cell death in both ABC and GCB DLBCL cells [41, 42]. In ABC DLBCL cells, DMF treatment induces succination of IKK2 and JAK1, which reduces their enzymatic activity, thereby decreasing NF-kB and STAT3 signaling, respectively [41]. Both signaling pathways are required for the survival and proliferation of ABC DLBCL cells [60, 61]. In GCB DLBCL, DMF induces ferroptosis, largely due to high expression of arachidonate 5-lipoxygenase combined with low levels of GSH and GPX4 [40]. We demonstrate that PRMT5 is upregulated in both ABC and GCB DLBCL, as well as MCL [28]. These findings provide a rationale for co-targeting PRMT5 with its specific inhibitor and the ferroptosis inducer DMF in DLBCL and MCL.

We demonstrate that ATF5, beyond its established role in regulating ferroptosis, holds potential as a prognostic biomarker in DLBCL and MCL, particularly when analyzed in conjunction with MYC expression. Our findings support the rationale for evaluating ATF5 and MYC co-expression in clinical DLBCL and MCL cohorts to further validate their prognostic value and explore potential functional interactions. Notably, ATF5 has been implicated in promoting cell survival, proliferation, and differentiation across various malignancies, including T-cell lymphoma [62], acute myeloid leukemia [63], and several solid tumors [64–67]. These roles suggest that ATF5 may function as a context-dependent oncogene, potentially interacting with lineage-specific transcriptional programs and stress response pathways. Future research should aim to delineate the gene regulatory networks controlled by ATF5 in B-cell malignancies, and investigate whether ATF5 directly cooperates with MYC or other oncogenic drivers to promote tumor progression.

## Supplementary information

SUPPLEMENTAL MATERIAL

## Data Availability

The RNA‑sequencing dataset used in this study was generated and published previously by our group and is publicly available in the Gene Expression Omnibus (GEO) under the accession number GSE115136. All gene‑expression analyses and alternative splicing analyses performed in this study were based on this RNA‑seq dataset. Kaplan–Meier survival analyses were conducted using publicly accessible GEO datasets, including DLBCL (n = 553; accession GSE125966) and MCL (n = 123; accession GSE93291). All datasets used in this study can be freely accessed through the GEO repository.
