# Supplementary material for: PRMT5 inhibition sensitizes B-cell lymphoma cells to ferroptosis
Source: Leukemia. 2026 Apr 17;40(6):1247–60. doi: 10.1038/s41375-026-02932-3 (PMC13233303; doi:10.1038/s41375-026-02932-3)
Supplement: Supplementary file 1 — SUPPLEMENTAL MATERIAL [file 41375_2026_2932_MOESM1_ESM.pdf]

Supplemental Methods and Materials:

Vectors Used for Gene Engineering: Knockout, Knockdown, and Inducible Overexpression
Gene knockout cells (PRMT5 KO and Myc KO) were generated as described in our previous study (see reference 28 for details). Briefly, an inducible two-vector CRISPR–Cas9 system was employed. The pR-CMV-Cas9-2A-Hygro vector (Cellecta) was used to establish Cas9-expressing cells, while the pRSGT16-U6Tet-sg-HTS6C-CMV-TetRep-2A-TagRFP-2A-Puro vector (Cellecta) was used to generate sgRNAs targeting specific genes. For gene knockdown, the pLKO.5 vector was used to constitutively deliver PRMT5 shRNAs. Inducible knockdown of ATF5 was achieved using the pRSMX vector. To inducibly overexpress ATF5 and ATF4, the Retro-cmv-to-pG vector was applied. Gene expression was induced by treatment with a low dose of doxycycline (20 ng/ml).

Retroviral and lentiviral transduction
Retroviral and lentiviral particles were produced in HEK293T cells using polyethyleneimine (PEI; Polysciences) transfection according to the manufacturer's protocol. For retroviral packaging, inducible shRNA knockdown or retro-CMV overexpression constructs were co-transfected with pHI60 and EA3 plasmids at a 2:1:1 ratio. For lentiviral packaging, constitutive shRNA knockdown vectors were co-transfected with PAX2 and MD2.G plasmids at the same 2:1:1 ratio. Viral supernatants were collected at 48 and 72 hours post-transfection, centrifuged to remove cellular debris, filtered through a 0.45 μm PVDF membrane, aliquoted, and stored at –80°C. When necessary, viral particles were concentrated by ultracentrifugation at 25,000 rpm for 2 hours, and pellets were resuspended in cold PBS. DLBCL and MCL cell lines were transduced with viral supernatants in the presence of 8 μg/mL polybrene (Sigma-Aldrich) by spin-infection at 2,500 rpm for 1 hour, followed by selection with appropriate antibiotics for 7 days to establish stable cell lines.

Lipid peroxidation assay
Following the indicated treatments, 1×10^6 cells per condition were treated with the specified concentrations of dimethyl fumarate (DMF) or solvent control for 3 hours. Cells were then washed once with pre-warmed Hank's Balanced Salt Solution (HBSS) and incubated with 1 μM BODIPY™ 581/591 C11 (Invitrogen) in HBSS for 20 minutes at 37°C. After staining, cells were washed twice with HBSS and immediately analyzed by flow cytometry. Just prior to acquisition, 10 ng/μL DAPI was added to each sample to exclude non-viable cells. Fluorescence was measured using a ThermoFisher Attune flow cytometer, and data were analyzed using FlowJo software (version 10.0).

Measurement of intracellular GSH and GSSG levels
Suspension cells were treated with the indicated compounds, harvested by centrifugation, and resuspended in phosphate-buffered saline (PBS). Intracellular levels of reduced glutathione (GSH) and oxidized glutathione (GSSG) were quantified using the GSH/GSSG-Glo™ Assay Kit (Promega, Cat# V6611) according to the manufacturer's instructions. Briefly, cells were transferred to white opaque 96-well plates, and two parallel sets were prepared to measure total glutathione (GSH + GSSG) and GSSG alone. Following cell lysis and reagent incubation, luminescence was measured using a plate reader. GSH levels were calculated by subtracting GSSG from total glutathione, and the GSH:GSSG ratio was determined. All values were normalized to total protein concentration.

Cell viability assay
Cell viability was assessed using the CellTiter-Glo® Luminescent Cell Viability Assay (Promega) according to the manufacturer's instructions. Briefly, cells were seeded at 4,000 cells per well in white opaque 96-well plates and treated with single agents or the indicated drug combinations for 72 hours. Luminescence, which is proportional to intracellular ATP levels, was measured as an indicator of

metabolically active (viable) cells. Drug synergy was quantified using SynergyFinder 2.0 (Ianevski et al., *Nucleic Acids Res.* 2020; PMID: 35713562) applying the HSA (Highest Single Agent) model, which compares the observed combination effect to the most active single agent. A synergy score greater than 0 indicates a synergistic interaction, and scores above 10 are generally considered significant. For each drug pair, the mean synergy score across the dose-response matrix was reported, with a more intense red color representing a higher synergy score. Interactive synergy distribution plots and summary synergy scores were generated for visualization. The half-maximal inhibitory concentration (IC_{50}) values were calculated using a four-parameter nonlinear regression model in GraphPad Prism (version 9.0).

Dual-Luciferase reporter gene assay

Dual-luciferase reporter assays were performed in triplicate at room temperature. HEK293T cells were co-transfected using polyethyleneimine (PEI; Polysciences) with 100 ng of pGL3-basic or various promoter constructs. For SLC7A11 promoter analysis, constructs included pGL3-Full-WT, pGL3-Full-ΔAAREs, pGL3-AAREs-WT, pGL3-ΔAAREs, and pGL3-UP-ve. For ATF4 promoter analysis, constructs included pGL3-Full, pGL3-Fr-1, and pGL3-Fr-2. For ATF5 promoter analysis, constructs included pGL3-F1, pGL3-F2, and pGL3-F3. Each reporter construct was co-transfected with 200 ng of either retro-CMV-To-pG (empty vector), retro-CMV-To-pG-OE-ATF5, retro-CMV-To-pG-OE-ATF4, MSCV-Myc-IRES-GFP, or combinations thereof, as indicated. A CMX-β-galactosidase expression vector (15 ng) was included in each transfection to normalize for transfection efficiency.
Forty-eight hours post-transfection, cells were harvested and lysed in buffer containing 0.1 M potassium phosphate (pH 7.8), 0.2% Triton X-100, 0.5 mM dithiothreitol (DTT), and 1 mg/mL bovine serum albumin (BSA; Fraction V, Sigma-Aldrich, Cat# A3059). Firefly luciferase activity was measured using the Luciferase Assay System (Promega), and β-galactosidase activity was measured using the Chemiluminescent Reporter Gene Assay System (Applied Biosystems). Firefly luciferase activity was normalized to β-galactosidase activity to account for transfection efficiency.

Chromatin immunoprecipitation (ChIP)

ChIP assays were performed as previously described with minor modifications (Liu, Blood 2023; PMID: 37738652). Briefly, 60 x 10^6 cells were fixed with 1% formaldehyde for 10 min at room temperature. The crosslinking reaction was stopped by addition of 1× glycine solution for 5 min. Cells were washed twice with ice-cold PBS. Cytosols were removed using ChIP cell lysis buffer. Nuclear pellets were resuspended in micrococcal nuclease buffer (MNase), treated with 112.5 unit of MNase for 15 min at 37°C and stopped by adding EDTA to the final concentration of 0.5M. Nuclear pellets were then washed once with MNase buffer, lysed with nuclear lysis buffer for 20 min at 4°C, and sonicated for a total of 5 min (5s on, 20s off, 20% amplitude, qsonica #Q500-110) at 4°C. 2% of fragmented DNA was kept as input. The remaining DNA fragments were recovered using anti-Myc (ab32072) or anti-HA-tag (ab9110) (1 µg for 60 million cells), or normal rabbit anti-IgG. DNA fragments were washed and reverse crosslinked as previously described. Recovered DNA fragments were purified using QIAGEN PCR purification kit and diluted in 500 µL of water. For each real-time PCR reaction, 3 µL was used with specific primers. The SensiFAST™ SYBR® Hi-ROX Kit (Bioline BIO-92020) was used for qPCR.

Alternative splicing analysis

To identify alternative splicing events (ASEs), RNA-seq alignment files (BAM format) were analyzed using rMATS v4.0.2 (replicate Multivariate Analysis of Transcript Splicing). The analysis focused on five major classes of splicing events: skipped exon (SE), mutually exclusive exons (MXE), alternative 3′ splice site (A3′SS), alternative 5′ splice site (A5′SS), and retained intron (RI). Comparisons were performed between PRMT5 knockout and control samples using the GRCh38 reference genome.

Significant ASEs were defined using the following stringent criteria: (1) absolute difference in exon inclusion level ($|\Delta\psi|$) ≥ 0.05; (2) false discovery rate (FDR) < 0.05; and (3) --cstat set to 0.01. To visualize representative splicing changes, rmats2sashimiplot was employed to generate sashimi plots.

Trypan Blue staining

Cell viability was measured with an automatic cell counter according to the manufacturer's instruction. Cells were suspended and mixed with equal volume of 0.4% trypan blue in PBS. Ten microliters of the cell suspension were loaded onto Bio-Rad TC20 system counting slides, and the number of viable cells was quantified on a TC20 automated cell counter (Bio-Rad).

Reverse transcription quantitative PCR (RT-qPCR)

Total RNA was extracted using the Monarch® Spin RNA Isolation Kit (New England Biolabs) according to the manufacturer's instructions and quantified using a NanoDrop Lite spectrophotometer (Thermo Scientific). For each sample, 1.5 µg of total RNA was reverse transcribed into cDNA using the First-Strand cDNA Synthesis Kit (Invitrogen). Quantitative PCR (qPCR) was performed using the SensiFAST™ SYBR® Hi-ROX Kit (Bioline, Cat# BIO-92005) on a CFX Connect Real-Time PCR Detection System (Bio-Rad). The thermal cycling conditions were as follows: initial denaturation at 95°C for 2 minutes, followed by 40 cycles of 95°C for 5 seconds and 60°C for 30 seconds. Gene expression levels were normalized to β-actin mRNA expression.

Immunoblotting analysis

Cell pellets were lysed in RIPA buffer (10 mM Tris-HCl, pH 8.0, 140 mM NaCl, 1 mM EDTA, 0.5 mM EGTA, 1% Triton X-100, 0.1% sodium deoxycholate, 0.1% SDS) supplemented with protease and phosphatase inhibitors (Thermo Fisher Scientific) on ice. Lysates were sonicated for 2 minutes (10 seconds on, 20 seconds off) and clarified by centrifugation at 15,000 × g for 20 minutes at 4°C. Protein concentrations were determined using the BCA Protein Assay Kit (Pierce). Equal amounts of protein (25 µg) were denatured in SDS sample buffer containing 2.5% β-mercaptoethanol at 95°C for 5 minutes. Samples were resolved on 4–20% RunBlue SDS-PAGE gels (CBS Scientific) and transferred to polyvinylidene difluoride (PVDF) membranes (Millipore, Cat# IPFL00010). Protein bands were visualized and quantified using the Odyssey Imaging System (LI-COR Biosciences).

Mice.

Male and female NOD.Cg-Prkdcscid Il2rgtm1Wjl/SzJ (NSG) breeder pairs were purchased from The Jackson Laboratory (Bar Harbor, ME, USA) and bred under specific pathogen-free conditions in sterile ventilated racks in the animal care facility at the University of Wisconsin-Madison. Both male and female mice were used between 8-16 weeks of age. The study was approved by the Animal Care and Use Committee of the University of Wisconsin-Madison (M005915).

Cell line-derived xenograft model

DLBCL OCI-Ly7 PRMT5 sgRNA cells were inoculated subcutaneously into the flank of male and female NSG mice. When tumors reached approximately 100 mm^3, mice were randomized and administered vehicle, DMF, doxycycline or the combination of both at the indicated doses in the figure or main text. Body weight and tumor diameter were measured twice a week, and tumor volume was determined by calculating the volume of an ellipsoid using the formula length × $width^2$ × 0.5. All values were expressed as mean ± standard error of the mean (SEM). When mice became moribund or when tumor size exceeded 20 mm in any direction, mice were euthanized as required by institutional protocols.

Drug formulation

For *in vivo* studies, doxycycline was administered in drinking water at a concentration of 2 mg/mL. Dimethyl fumarate (DMF) was formulated in 2.5% DMSO, 15% PEG400, 2.5% Tweens 80, and 80% water. GSK3326595 was formulated in 5.0 % DMSO, and 95% water. All compounds were freshly prepared and administered immediately after formulation.

Immunohistochemistry (IHC) Staining and Quantification

Tumor tissues harvested from NSG mice were fixed in formalin and embedded in paraffin following euthanasia. Immunohistochemical staining was performed by the Experimental Animal Pathology Laboratory at the Carbone Cancer Center (Madison, WI). The following primary antibodies were used: rabbit anti-cleaved Caspase-3 (Cell Signaling Technology, #9664S; 1:250), PRMT5 (Abcam, ab31751; 1:400), and 4-HNE (Abcam, ab48506; 1:100). For each experimental group, 6–7 tumor samples were processed for staining. Four representative images were captured from each stained sample. Protein expression levels were quantified using Fiji (ImageJ) software.

REAGENT or RESOUECE	SOURCE
**Antibodeis**	
Rabbit polyclonal anti-PRMT5 antibody (ab31751)	Abcam
Mounse monoclona anti-4 Hydroxynonenal antibody [HNEJ-2]	Abcam
Rabbit monoclona anti-ATF5 antibody	Santa Cruz
Rabbit monoclona anti-ATF4 antibody	Cell signaling
Rabbit monoclona anti-xCT/SLC7A11 (D2M7A)	Cell signaling
Rabbit polyclonal anti-AIFM2/FSP1 antibody	Cell signaling
Rabbit monoclona anti-Cleaved Caspase-3 (Asp175)	Cell signaling
Mouse monoclonal anti-Glutathione Peroxidase 4/GPX4 Antibody (E-12)	Santa cruz
Rabbit monoclonal anti-c-Myc antibody [Y69]	Abcam
Mouse monoclonal anti-GSK-3β (3D10) antibody	Cell signaling
Rabbit polyclonal anti-MEP50 antibody	Cell signaling
Rabbit monoclonal anti-beta-Actin	Cell signaling
Mouse anti-α-Tubulin Mouse mAb (DM1A)	Sigma
Rabbit polyclonal anti-Histone H4 (symmetric di methyl R3) antibody	Abcam
Rabbit monoclonal anti-Histone H3	Cell signaling
Anti-rabbit IgG, HRP-linked Antibody	Cell signaling
Anti-mouse IgG, HRP-linked Antibody	Cell signaling
Normal mouse IgG	Santa Cruz
Normal mouse IgG1	Santa Cruz
Mouse monoclonal anti-FLAG® M2 antibody	Sigma-Aldrich
Mouse monoclona anti-HA Tag antibody (2-2.2.14)	Invitrogen
Rabbit polyclonal anti-HA tag antibody - ChIP Grade	Abcam
FITC mouse anti-human CD20 clone LT20	Miltenyi Biotec
PE mouse anti-human CD19 Clone 4G7	R&D biotechne
PE mouse IgG1 κ Isotype Control	BD biosciences
APC mouse anti-human CD38	BD biosciences
APC mouse IgG1 κ Isotype Control	BD biosciences

Biological Samples	
Primary MCL_4	The same sample was used in the study titled 'EGR1-mediated metabolic reprogramming to oxidative phosphorylatic
Primary MCL_9	The same sample was used in the study titled 'EGR1-mediated metabolic reprogramming to oxidative phosphorylatic

REAGENT or RESOUECE	SOURCE
**Chemicals and Critical Commercial assays**	
GSK3326595	Chemietek
EPZ015666	Cayman Chemical
DMF	Selkchem
RSL3	Target Mol
Erastin	Target Mol
Ferrostatin-1	Target Mol
AZD5356	Cayman Chemical
AKTi-V (Triciribine)	selleckchem
BODIPY™ 581/591 C11 (Lipid Peroxidation Sensor)	Thermo Fisher Scientific
Galacto-Star™ β-Galactosidase Reporter Gene Assay System	Thermo Fisher Scientific
SuperScript™ IV First-Strand Synthesis System	Thermo Fisher Scientific
SuperSignal™ West Femto Maximum Sensitivity Substrate	Thermo Fisher Scientific
Pierce™ BCA Protein Assay Kit	Thermo Fisher Scientific
Luciferase Assay System	Promega
CellTiter-Glo(R) 2.0 Assay	Promega
GSH/GSSG-Glo™ Assay	Promega
Dynabeads™ Protein G for Immunoprecipitation	Thermol Fisher Sci
Dynabeads™ Protein A for Immunoprecipitation	Thermol Fisher Sci
PI flow dye (BD)	BD pharmingen
Doxycycline hyclate	SIGMA-ALDRICH
RPMI-1640 medium (1x) -L-glutamine	HyClone
DMEM/High glucose with L-glutamine, sodium pyruvate	HyClone
FBS	HyClone
Pen/strep solution	lonza
Corning® glutagro™	Corning
MEM Non-Essential Amino Acid Solution (100X)	Lonza
Sodium Pyruvate Solution	GE Healthcare Life Sciences
Trypan Blue Solution (w/v) in PBS	corning
Protease inhibitor cocktail	sigma-aldrich
PMSF Protease Inhibitor	Thermo Scientific™
D-Luciferin, Sodium Salt	Gold Biotechnology
QIAquick PCR Purification kit	QIAGEN
RNeasy Plus Mini Kit	QIAGEN
Monarch® Spin RNA Isolation Kit (Mini)	NEB
In-Fusion® Snap Assembly Master Mix with Competent Cells	Takara
SensiFast SYBR Hi-Rox Kit	Bioline
PEG300	Selleck Chemical LLC
Triton X-100	EMD MILLIPORE
pierce 16% formaldehyde (w/v), methanol-free	pierce
PureLink™ RNase A	Invitrogen

Oligo Name	Sequence
Human SLC7A11 mRNA-For	TCTCCAAAGGAGGTTACCTGC
Human SLC7A11 mRNA-Rev	AGACTCCCCTCAGTAAAGTGAC
Human GCLC mRNA-For	GGAAGTGGATGTGGACACCAGA
Human GCLC mRNA-Rev	GCTTGTAGTCAGGATGGTTTGCG
Human ATF5 mRNA-For	GCTCGTAGACTATGGGAAACTCC
Human ATF5 mRNA-Rev	CATCCAGTCAGAGAAGCCATCAC
Human-ATF5 Flag-cDNA-mRNA-For	CG GGATCC GCCACC ATG GAC TAC AAA GAC GAT GAC GAC AAG TCACTCCTGGCGACC
Human-ATF5 cDNA-mRNA-For	CG GGATCC GCCACC ATG TCACTCCTGGCGACC
Human-ATF5 cDNA-mRNA-Rev	CC CTCGAG CTAGCAGCTACGGGTCCT
Human-ATF5-cDNA-BamH1-For	AATTTAAATCGGATCCGCCACCATGTCACTCCTGGCGACC
Human-ATF5-cDNA-HA-Not1-Rev	CAGATCCTTGCGGCCGCCTAAGCGTAATCTGGAACATCGTATGGGTAGCAGCTACGGGTCCTCTG
Human-ATF4 cDNA-mRNA-For	CGGGATCCGCCACCATGTTGGAGAAAATGGATTT
Human-ATF4 cDNA-mRNA-Rev	CC CTCGAG CTAGGGGACCCTTTTCTT
Human-SLC7A11 cDNFA-mRNA-For	CCGCTCGAGCCATTAAGTTTCCTTGTTGTACTGTTACTTCC
Human-SLC7A11 cDNFA-mRNA-Rev	CCCAAGCTTCTGACGGTGGGTAAACAAACCG
pLKO.5-PRMT5 shRNA#1	TRCN0000303446 (GGCTCAAGCCACCAATCTATG)
pLKO.5-PRMT5 shRNA#2	TRCN0000303447 (CCCATCCTCTTCCCTATTAAG)
pLKO.5-PRMT5 shRNA#3	TRCN0000299130 (GCCCAGTTTGAGATGCCTTAT)
pLKO.5 control sh	SHC202
human SLC7A11 promoter- AAREs-WT For	TCTTACGCGTGCTAGGCTTTGTTCTCTAAAAAGCTTAGG
human SLC7A11 promoter- AAREs-WT Rev	GATCGCAGATCTCGATCCACCTCCTCGTTCCACC
human SLC7A11 promoter- UP-ve For	TCTTACGCGTGCTAGTTTGGTAATTCAAAGGAACAAC
human SLC7A11 promoter- UP-ve Rev	GATCGCAGATCTCGACAAAATTAAAGATTCTACACTTTAT
human SLC7A11 promoter- Full WT	GenScript gene fragements synthesis
human SLC7A11 promoter- ΔAAREs	GenScript gene fragements synthesis
Human ATF5 promoter -F1-For	CCGCTCGAGGATTCTTCGTGCTTGGTTGT
Human ATF5 promoter -F1-Rev	CCCAAGCTTGGTTCTATCCCCACCCACTT
Human ATF5 promoter -F2-For	CCGCTCGAGCCCGGCCATGGCTCTGTA

Human ATF5 promoter -F2-Rev	CCCAAGCTTCCCCGGGCCCAGTGCGCA
Human ATF5 promoter -F3-For	CCGCTCGAGCCTGAATGGCCGCACATG
Human ATF5 promoter -F3-Rev	CCCAAGCTTGGTGCCTGGTTTAAAAGGAAG
Human ATF4 promoter -Fr1-For	CCGCTCGAGACTAGCAGCGACCCGAGA
Human ATF4 promoter -Fr1-Rev	CCCAAGCTTCGAAAGGAGAGAGGTGTACTAC
Human ATF4 promoter -Fr2-For	CCGCTCGAGCGTGAGGCCATAAGAACAAAC
Human ATF4 promoter -Fr2-Rev	CCCAAGCTTGGGAGGAGGGAAAACGCA
Human shRNA control duplex	GATCCC GTGGATTTCGAGTCGTCTTAAT TCAAGAGATTAAGACGACTCGAAATCCAC TTTTT<br>AGCTAAAAA GTGGATTTCGAGTCGTCTTAAT CTCTTGAATTAAGACGACTCGAAATCCAC GG
Human shATF5#1 duplex	GATCCC TATCACCTCTCTTGCGTATTT TTCAAGAGA AAATACGCAAGAGAGGTGATA TTTTT<br>AGCTAAAAA TATCACCTCTCTTGCGTATTT TCTCTTGAA AAATACGCAAGAGAGGTGATAGG
Human shATF5#2 duplex	GATCCC ATGTCTATGCCCGTCACATAA TTCAAGAGA TTATGTGACGGGCATAGACAT TTTTT<br>AGCTAAAAA ATGTCTATGCCCGTCACATAA TCTCTTGAA TTATGTGACGGGCATAGACAT GG

**Software and Algorisms**	
ImageJ 142 software	NIH
GraphPad Prism 9.0	Graphpad
FlowJo Software vX 10.0.7v2	FLOWJO, LLC
GSEA v3.0	Broad Institute
Igtegrative Gomomic Viewer v2.3.91(145)	Broad Institute

Supplemental Figures and Figure Legends

Supplemental Figure 1

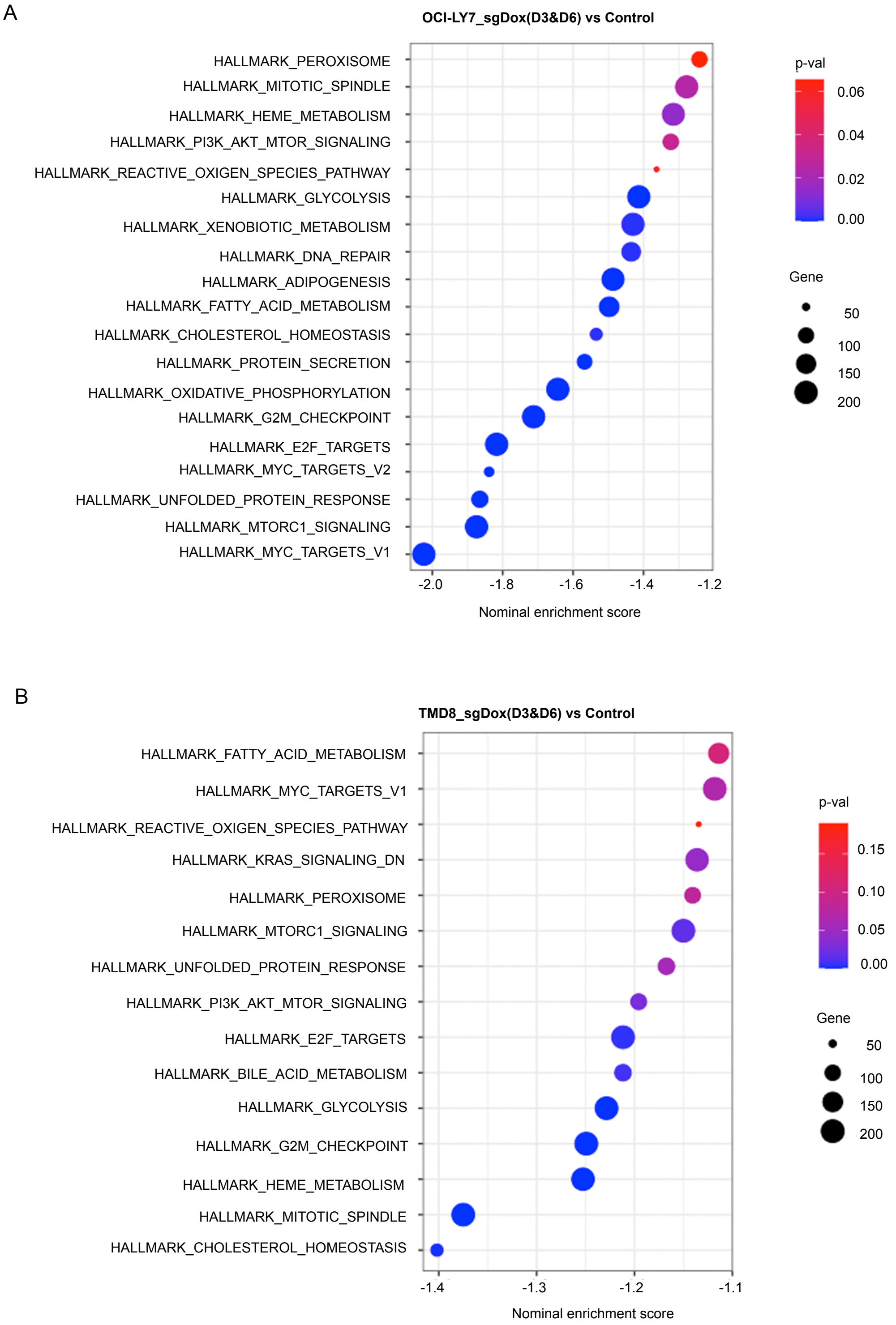

Supplemental Figure 1. Pathway enrichment analysis of RNA-seq data in PRMT5 knockout cells. (**A–B**) Ranked lists of the most significantly enriched pathways based on RNA-seq data (GSE115136) comparing PRMT5 knockout and control cells. (**A**) OCI-Ly7 PRMT5 knockout cells (*n* = 4) versus control cells (*n* = 2). (**B**) TMD8 PRMT5 knockout cells (*n* = 4) versus control cells (*n* = 2).

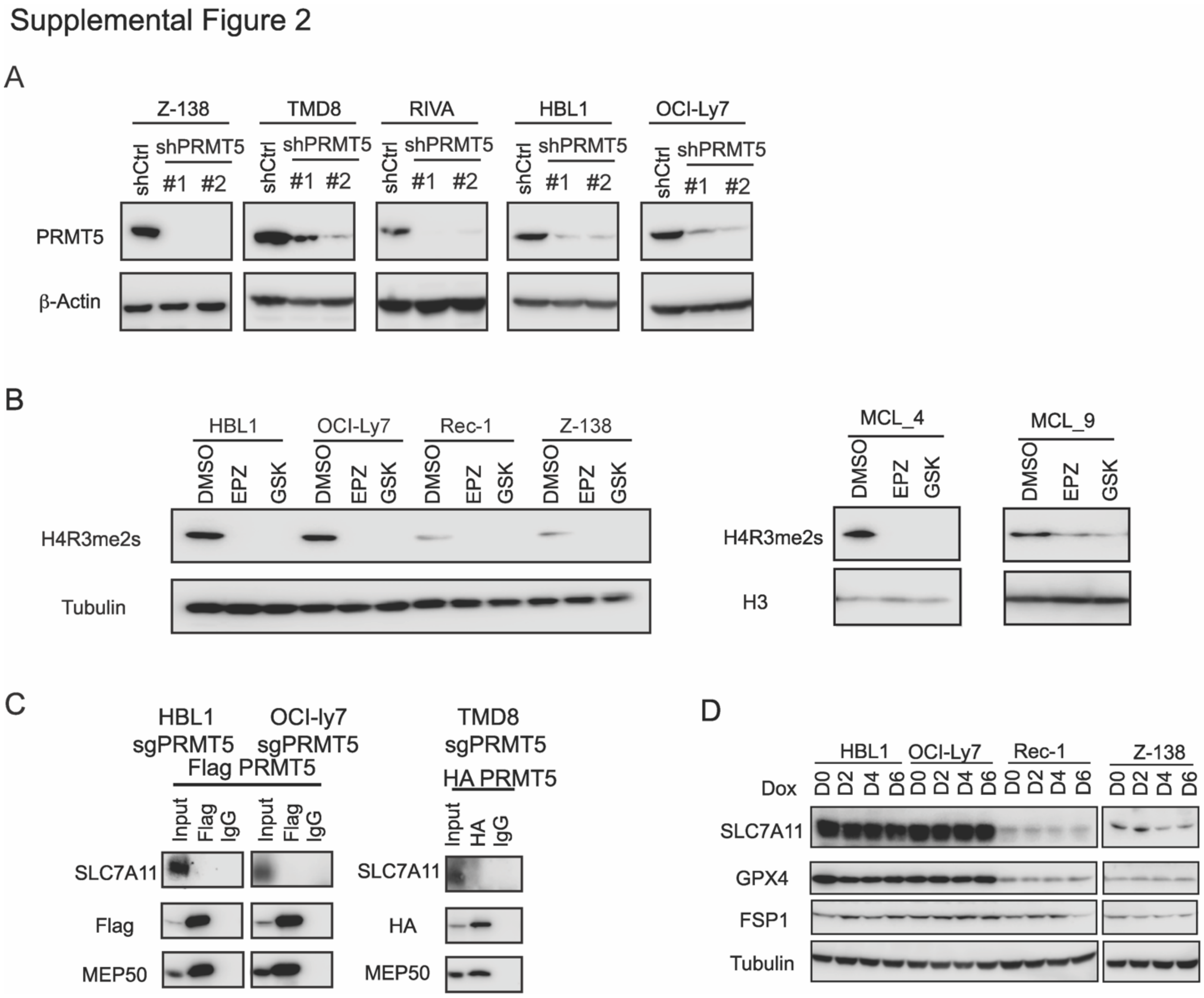

Supplemental Figure 2. Validation of PRMT5 knockdown and inhibition efficiency in B-Cell lymphoma cell lines and patient samples. (**A**) Immunoblot analysis of PRMT5 expression in various PRMT5 knockdown cell lines, including mantle cell lymphoma (MCL) line Z-138, and the diffuse large B-cell lymphoma (DLBCL) subtypes: activated B-cell–like (ABC) TMD8, RIVA, HBL1, and germinal center B-cell–like (GCB) OCI-Ly7. β-Actin was used as a loading control. (**B**) Immunoblot analysis of H4R3me2s expression in MCL and DLBCL cell lines treated with PRMT5 inhibitors EPZ015666 (1 µM) or GSK3326595 (1 µM) for 5 days. Tubulin served as a loading control (left panel). Right panel: H4R3me2s expression in primary MCL patient samples treated with the same inhibitors for 5 days; H3 served as a loading control. (**C**) Co-immunoprecipitation (Co-IP) analysis reveals no detectable interaction between PRMT5 and SLC7A11 in HBL1, OCI-Ly7, and TMD8 cells exogenously expressing FLAG-PRMT5 or HA-PRMT5 in the absence of endogenous PRMT5. MEP50 was included as a positive control for PRMT5-binding proteins. Protein interactions were assessed by immunoblotting. **(D)** Immunoblot analysis of SLC7A11, GPX4, and FSP1 expression in DLBCL cell lines (HBL1 and OCI-Ly7) and MCL cell lines (Rec-1 and Z-138) treated with a low dose of doxycycline (20 ng/mL) for the indicated durations (0, 2, 4, and 6 days). Tubulin was used as a loading control.

Supplementary Figure 3

A

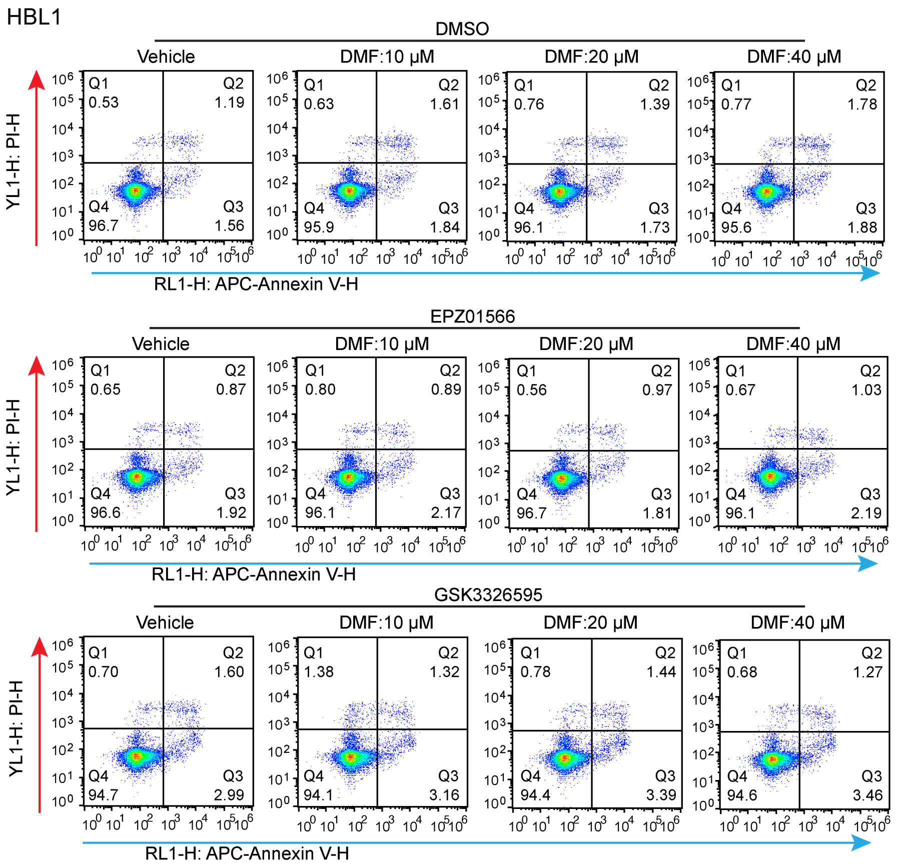

B

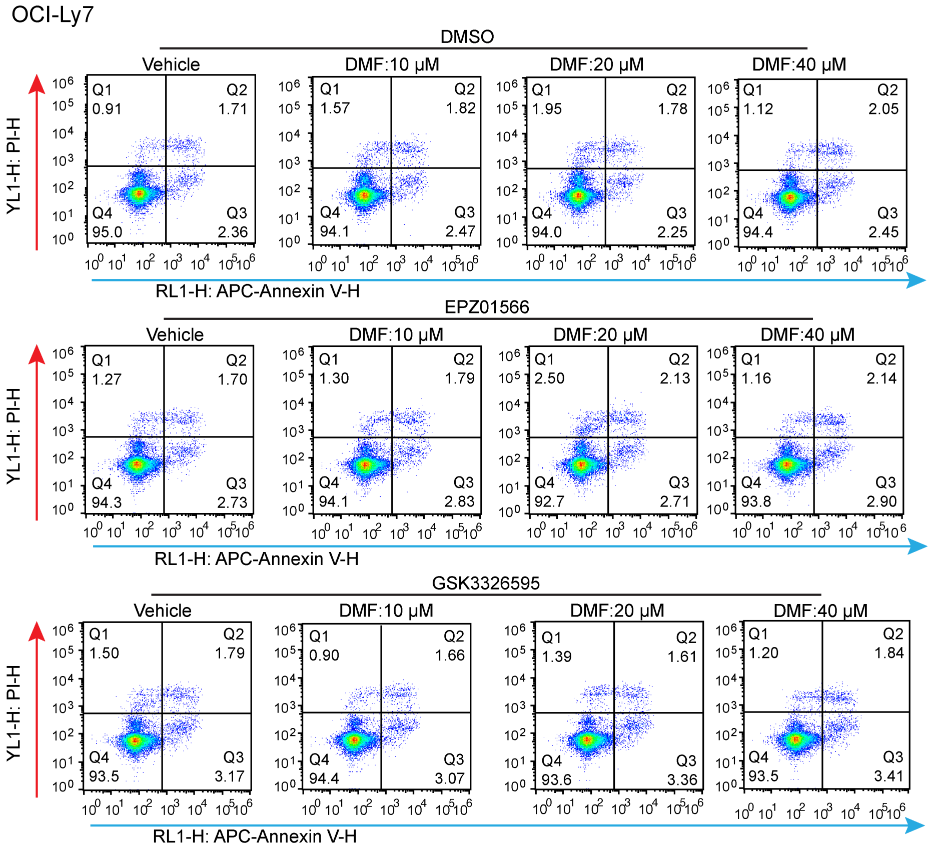

Supplementary Figure 3 Continue

C

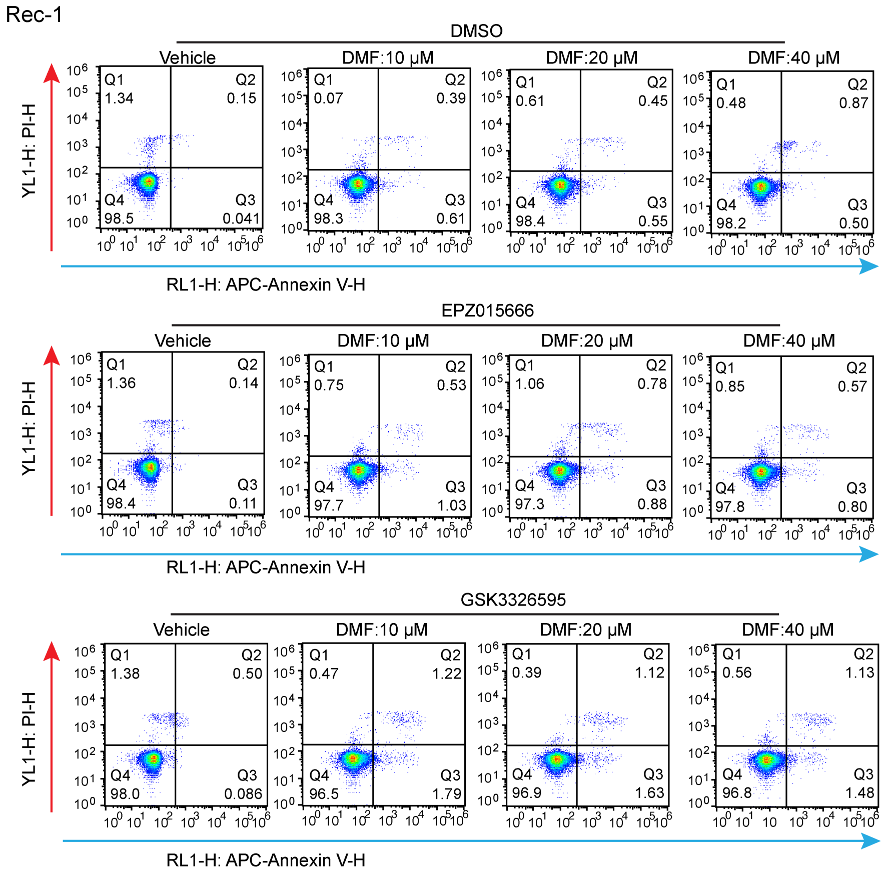

D

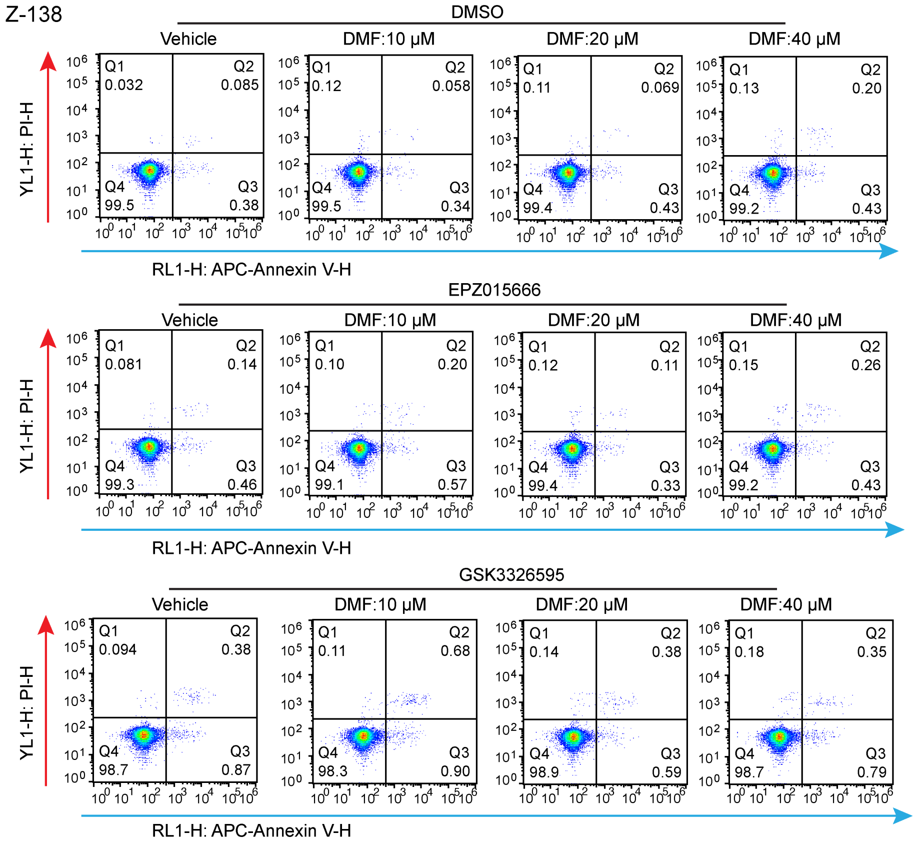

Supplemental Figure 3. Short-term DMF treatment does not induce apoptosis in DLBCL and MCL cells. (A–D) Flow cytometric analysis of apoptosis in DLBCL cell lines (A and B) and MCL cell lines (C and D) treated with PRMT5 inhibitors GSK3326595 (1 μM) or EPZ015666 (1 μM), or vehicle control, for 5 days, followed by a 3-hour treatment with increasing concentrations of DMF (0, 10, 20, and 40 μM).

Supplemental Figure 4

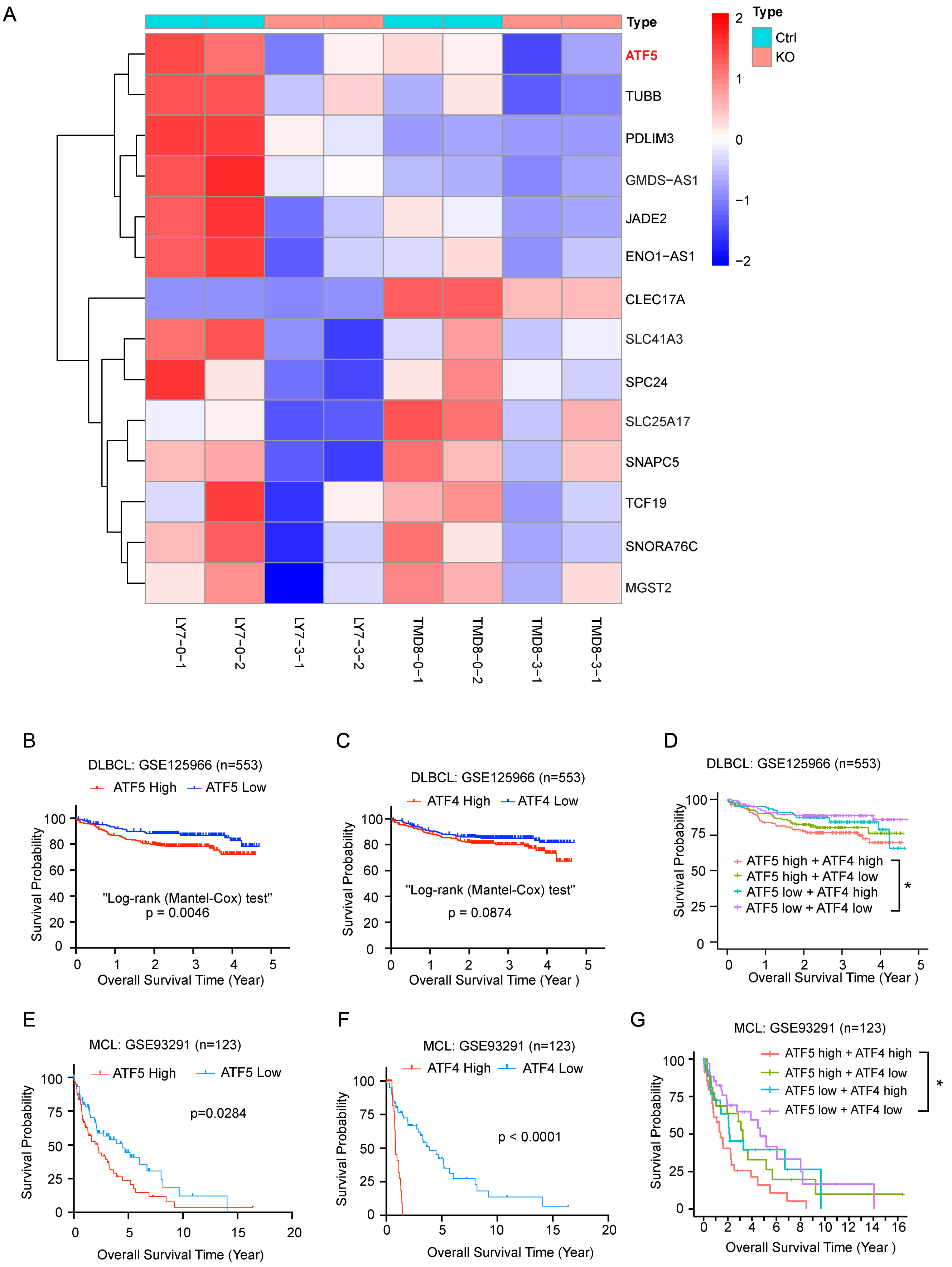

Supplemental Figure 4. Top dysregulated genes following PRMT5 knockout and survival analyses of ATF5 and ATF4 in DLBCL and MCL patient samples. (**A**) Heatmaps showing the top differentially expressed genes between PRMT5 knockout and control cells in OCI-Ly7 and TMD8 cell lines. PRMT5 knockout was induced with doxycycline for 3 days (n = 2 per group). (**B–C**) Kaplan–Meier analysis of overall survival in patients with DLBCL (n = 553, GSE125966), stratified by expression levels of ATF5 (B) and ATF4 (C). High expression of ATF5 is associated with poor overall survival. (**D**) Kaplan–Meier survival curves of DLBCL patients grouped by combined ATF5 and ATF4 expression levels. Patients were categorized into four groups based on whether each gene's expression was above or below the median ("high" or "low"). Co-high expression of ATF5 and ATF4 is associated with poorest overall survival. (**E–F**) Kaplan–Meier analysis of overall survival in patients with MCL (n = 123, GSE93291), stratified by expression levels of ATF5 (E) and ATF4 (F). High expression of either gene correlates with worse overall survival. (**G**) Kaplan–Meier survival curves of MCL patients grouped by combined ATF5 and ATF4 expression levels, categorized as described in (D). Co-high expression of ATF5 and ATF4 is associated with significantly poorer overall survival outcomes. Survival differences were evaluated using the log-rank (Mantel–Cox) test; overall p values are shown. Pairwise comparisons were performed using pairwise log-rank tests. Confidence intervals are not shown.

Supplemental Figure 5

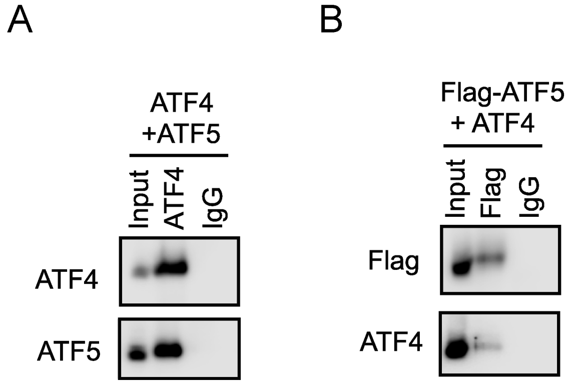

Supplemental Figure 5. ATF4 interacts with ATF5. (**A**) Co-immunoprecipitation (Co-IP) using an anti-ATF4 antibody demonstrates a physical interaction between ATF4 and ATF5, as detected by immunoblot analysis. HEK293T cells were co-transfected with 2 μg each of ATF4 and ATF5 overexpression plasmids. Forty-eight hours post-transfection, cell lysates were collected and subjected to immunoblotting. (**B**) Co-IP using an anti-Flag antibody confirms the interaction between ATF4 and Flag-tagged ATF5. HEK293T cells were co-transfected with 2 μg of ATF4 and 2 μg of Flag-ATF5 plasmids. Lysates were collected 48 hours post-transfection and analyzed by immunoblotting.

Supplemental Figure 6

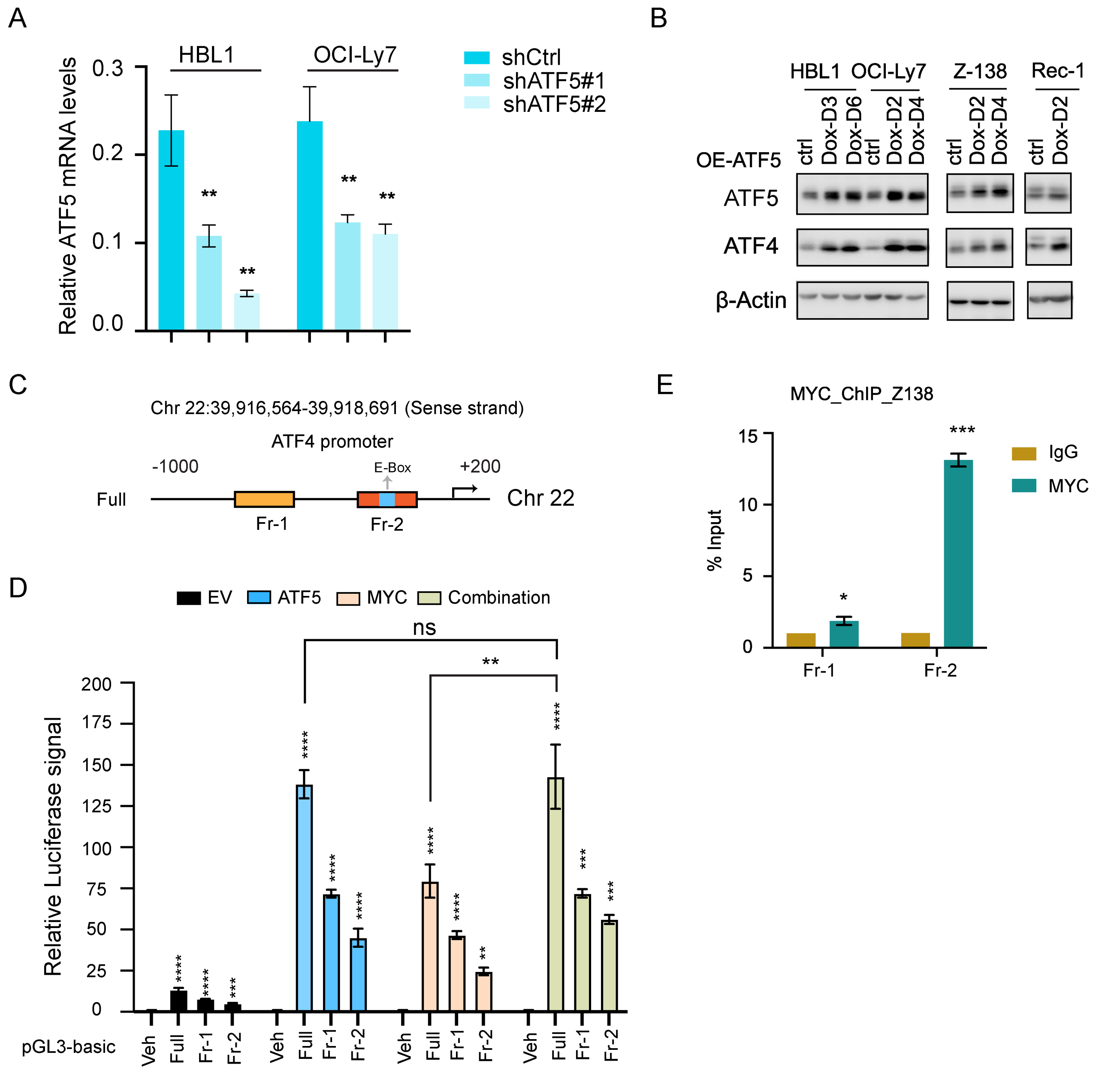

Supplemental Figure 6. ATF5 regulates ATF4 expression at the transcriptional level.
(**A**) Quantitative real-time PCR analysis showing reduced mRNA expression of ATF5 relative to β-actin in HBL1 and OCI-Ly7 cells following doxycycline (Dox)-inducible ATF5 shRNA knockdown (20 ng/mL for 4 days). Data represent mean ± SD from three biological replicates (**p < 0.01, n = 3). (**B**) Immunoblot analysis showing increased ATF5 and ATF4 protein levels following Dox-inducible overexpression of ATF5. Cells were treated with 20 ng/mL Dox for the indicated durations prior to analysis. (**C**) Schematic of ATF4 promoter constructs used in luciferase reporter assays. The full-length construct spans 1,200 bp (–1000 to +200 relative to the transcription start site [TSS]). Fr-1 and Fr-2 represent truncated promoter fragments. (**D**) Dual-luciferase reporter assay in HEK293T cells showing that ATF5 or Myc individually activate ATF4 promoter activity. Co-expression of ATF5 and Myc did not significantly enhance activity compared to ATF5 alone. Firefly luciferase activity (pGL3-basic) was normalized to β-galactosidase. Data represent mean ± SD from three independent experiments (one-way ANOVA, **p < 0.01, ***p < 0.001, ****p < 0.0001; n = 3). (**E**) MYC ChIP-

qPCR analysis using two primer sets targeting the ATF4 promoter and TSS regions. IgG served as a negative control. Data represent mean ± SD (*p < 0.05, ***p < 0.001; n = 3).

Supplemental Figure 7

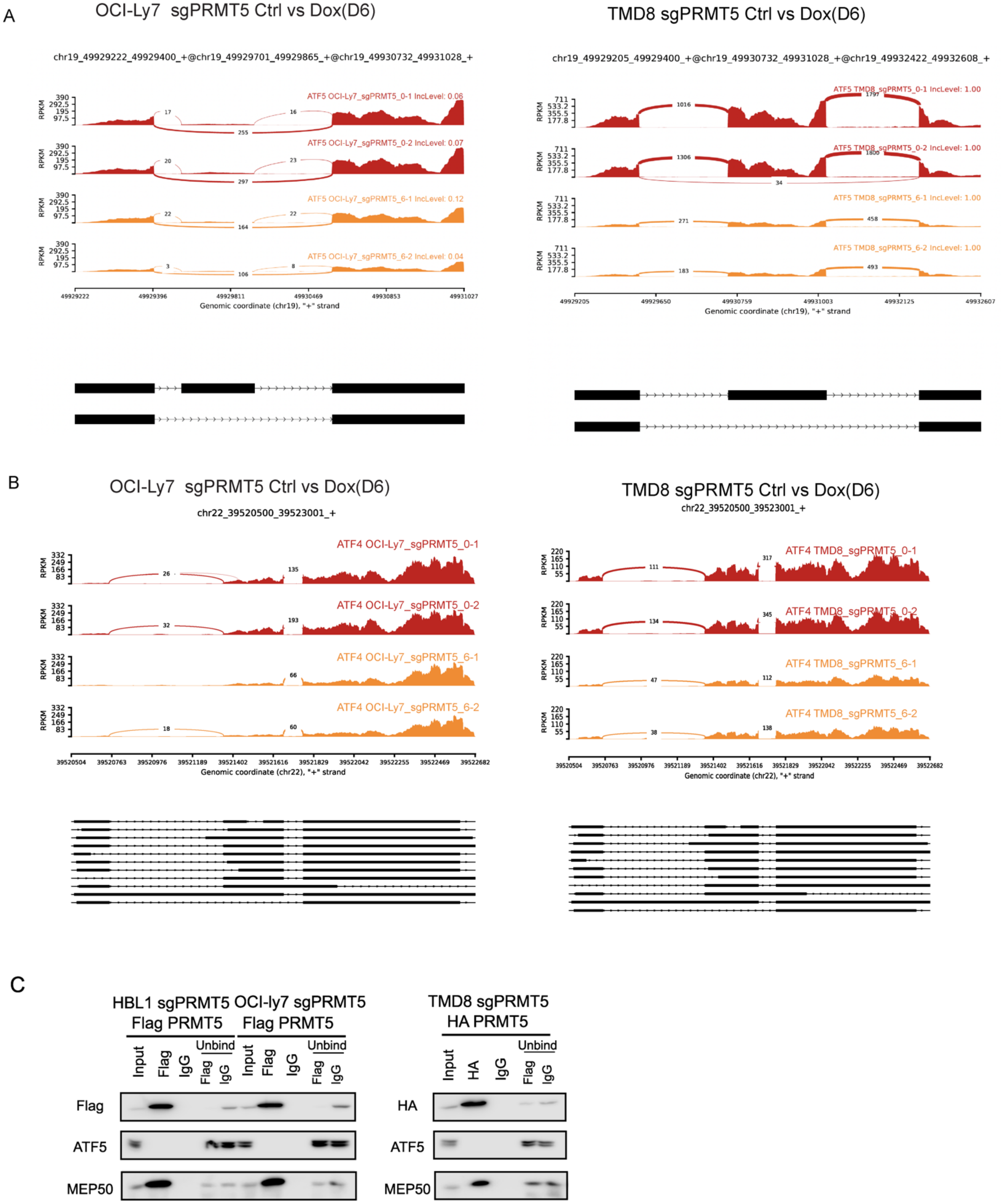

Supplemental Figure 7. PRMT5 does not regulate ATF5 splicing or physically interacts with ATF5. (**A**) PRMT5 knockout does not alter ATF5 splicing in OCI-Ly7 and TMD8 cells. Sashimi plots display RNA-seq read coverage and exon–exon junctions at the *ATF5* locus in OCI-Ly7 (left) and TMD8 (right) cells under control and PRMT5 knockout (sgPRMT5) conditions following 6 days of doxycycline treatment (D6). Replicate tracks are shown for control (red) and sgPRMT5 (orange) samples. Junction read counts are indicated on arcs, and inclusion levels (IncLevel) for selected splicing events are shown to the right of each track. Genomic coordinates (hg38) are shown on the x-axis; read coverage (RPKM) is shown on the y-axis. The *ATF5* gene model is displayed below each plot. (**B**) PRMT5 knockout does not affect *ATF4* splicing in OCI-Ly7 and TMD8 cells. Sashimi plots show RNA-seq read coverage and exon–exon junctions at the *ATF4* locus in OCI-Ly7 (left) and TMD8 (right) cells under control and sgPRMT5 conditions after 6 days of doxycycline treatment. Replicate tracks for control (red) and sgPRMT5 (orange) samples are shown. Junction read counts and IncLevel values are annotated as in (A). Genomic coordinates (hg38) and read coverage (RPKM) are indicated on the x- and y-axes, respectively. The *ATF4* gene model is shown below each plot. (**C**) Co-immunoprecipitation (Co-IP) analysis reveals no detectable interaction between PRMT5 and ATF5 in HBL1, OCI-Ly7, and TMD8 cells exogenously expressing Flag-PRMT5 and HA-ATF5 in the absence of endogenous PRMT5. MEP50 was included as a positive control for PRMT5-binding proteins. Protein interactions were assessed by immunoblotting.

Supplemental Figure 8

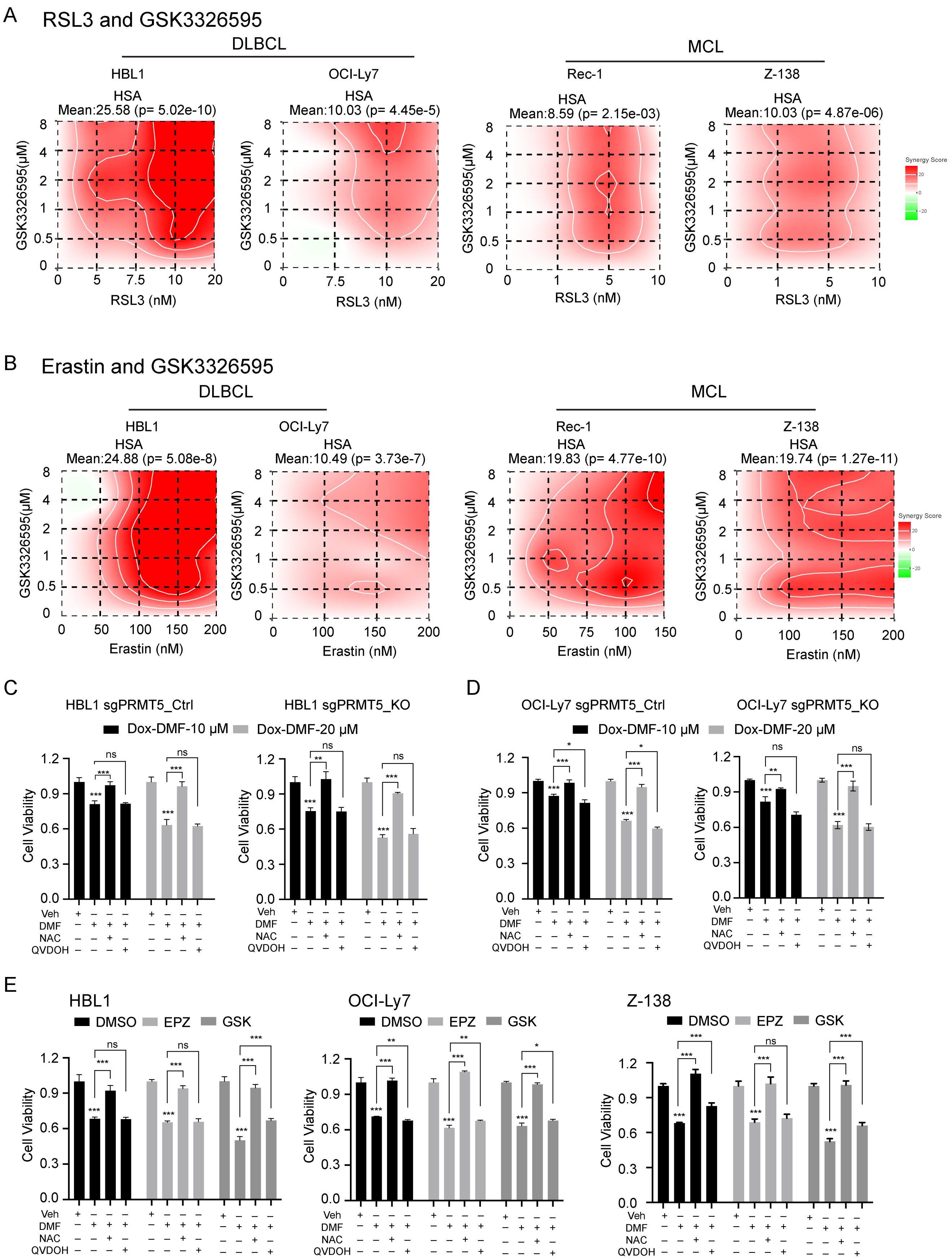

Supplemental Figure 8. The ferroptosis inducers RSL3 and Erastin exhibit synergistic effects with the PRMT5 inhibitor GSK3326595. (**A**) Synergy analysis of the PRMT5 inhibitor GSK3326595 in combination with the GPX4 inhibitor RSL3 in the indicated DLBCL (HBL1 and OCI-ly7) and MCL (Rec-1 and Z-138) cell lines. Synergy scores were calculated using SynergyFinder 2.0. A synergy score > 0 (pink) indicates a synergistic interaction between the two compounds. (**B**) Synergy analysis of GSK3326595 in combination with the SLC7A11 inhibitor Erastin in the indicated DLBCL (HBL1 and OCI-ly7) and MCL (Rec-1 and Z-138) cell lines. Synergy scores were calculated using SynergyFinder 2.0. A synergy score > 0 (pink) indicates a synergistic interaction between the two compounds. **(C–D)** The well-known antioxidant and glutathione precursor N-acetylcysteine (NAC) rescued the growth inhibition induced by DMF (15 μM) in both control and PRMT5 knockout HBL1 (C) and OCI-Ly7 (D) cells, as measured by the CellTiter-Glo™ Luminescent Cell Viability Assay. In contrast, the broad-spectrum apoptosis inhibitor Q-VD-OPh (QVDOH) did not rescue DMF-induced growth inhibition in these cell lines. **(E)** NAC also rescued DMF-induced growth inhibition following pretreatment with different PRMT5 inhibitors (EPZ015666 and GSK3326595) in HBL1 and OCI-Ly7 cells (15 μM DMF) and in Z-138 cells (10 μM DMF). QVDOH partially rescued DMF-induced growth inhibition in the same cell lines. Cell viability was assessed using the CellTiter-Glo™ Luminescent Cell Viability Assay. Error bars represent the mean ± SD from three biological replicates (*p < 0.05, **p < 0.01, ***p < 0.001).

Supplemental Figure 9

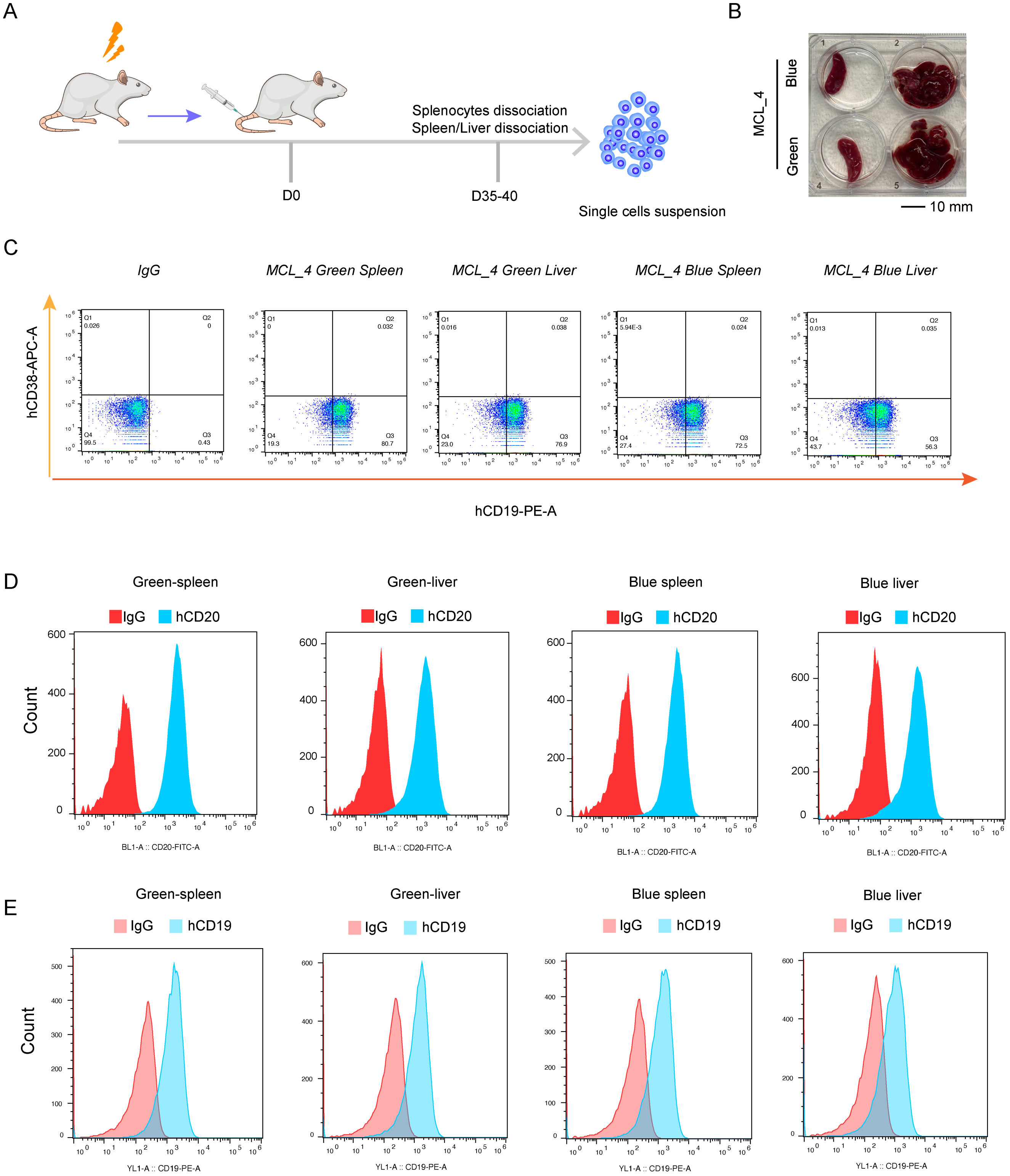

Supplemental Figure 9. Establishment and characterization of MCL patient-derived xenograft (PDX) models in NSG mice. (**A**) Schematic illustration of the expansion protocol for MCL patient-derived cells in NSG mice. A total of 2 × 10^6 MCL cells were resuspended in 0.1 mL PBS and injected via the tail vein into sublethally irradiated (2 Gy) NSG mice. After 35–42 days, spleens and livers were harvested to generate single-cell tumor suspensions. (**B**) Representative images of spleens and livers from MCL PDX mice. (**C**) Flow cytometric analysis of human CD19 (hCD19) and CD38 (hCD38)

expression in harvested MCL samples. (**D-E**) Flow cytometric analysis of human CD20 (hCD20)(D) and CD19 (hCD19)(E) expression in MCL cells collected from NSG mice.

Supplemental Figure 10

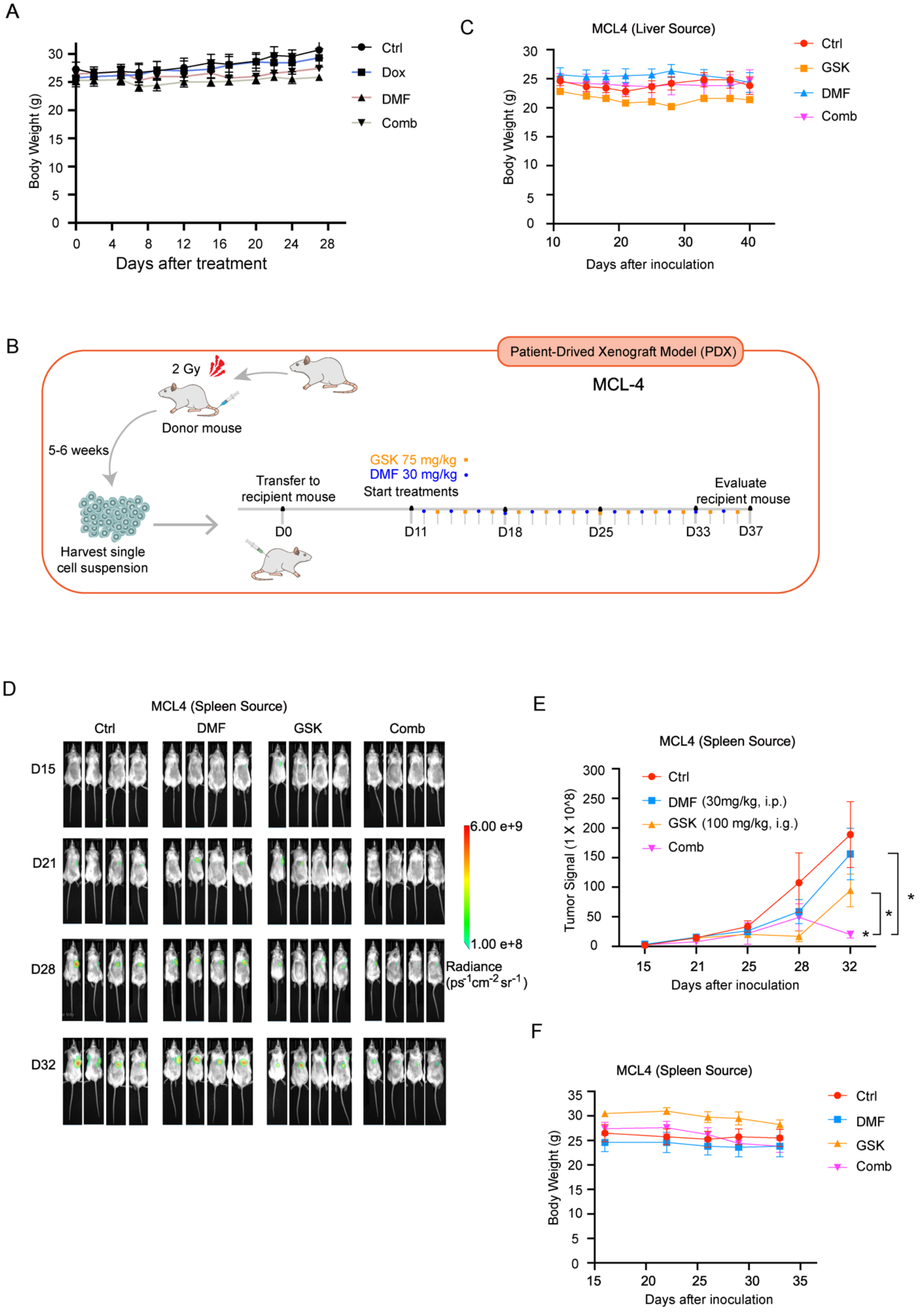

Supplemental Figure 10. Drug treatments do not induce significant body weight loss in MCL PDX models. (**A**) Body weight changes during treatment in the OCI-Ly7 sgPRMT5 xenograft model. Data are presented as mean ± SEM, showing no significant weight loss across treatment groups. **(B)** Schematic illustration of the patient-derived xenograft (PDX) model of mantle cell lymphoma (MCL) and the treatment protocol. (**C**) Body weight changes during treatment in the MCL-4 PDX model derived from liver tissue (C). (**D–E**) Establishment and treatment of an MCL PDX model. Freshly isolated MCL-4 cells from spleen tissue were subcutaneously inoculated into NSG mice. Tumor burden was monitored using the Lago X bioluminescence imaging system (Spectral Instruments Imaging). On Day 15 post-inoculation, mice were randomized into four treatment groups ($n = 4$). Starting on Day 16, mice received 100 mg/kg GSK3326595 (oral, every other day), 30 mg/kg DMF (intraperitoneal, every other day), or a combination of both for 16 days. Representative bioluminescence images are shown. The color scale indicates photon flux (photons/second) emitted by tumors. Error bars represent mean ± SEM (two-way ANOVA; $*p < 0.05$, $**p < 0.001$). (**F**) Body weight changes during treatment in each experimental group ($n = 4$). Data are presented as mean ± SEM, confirming no significant weight loss during the treatment period.

Supplemental Figure 11

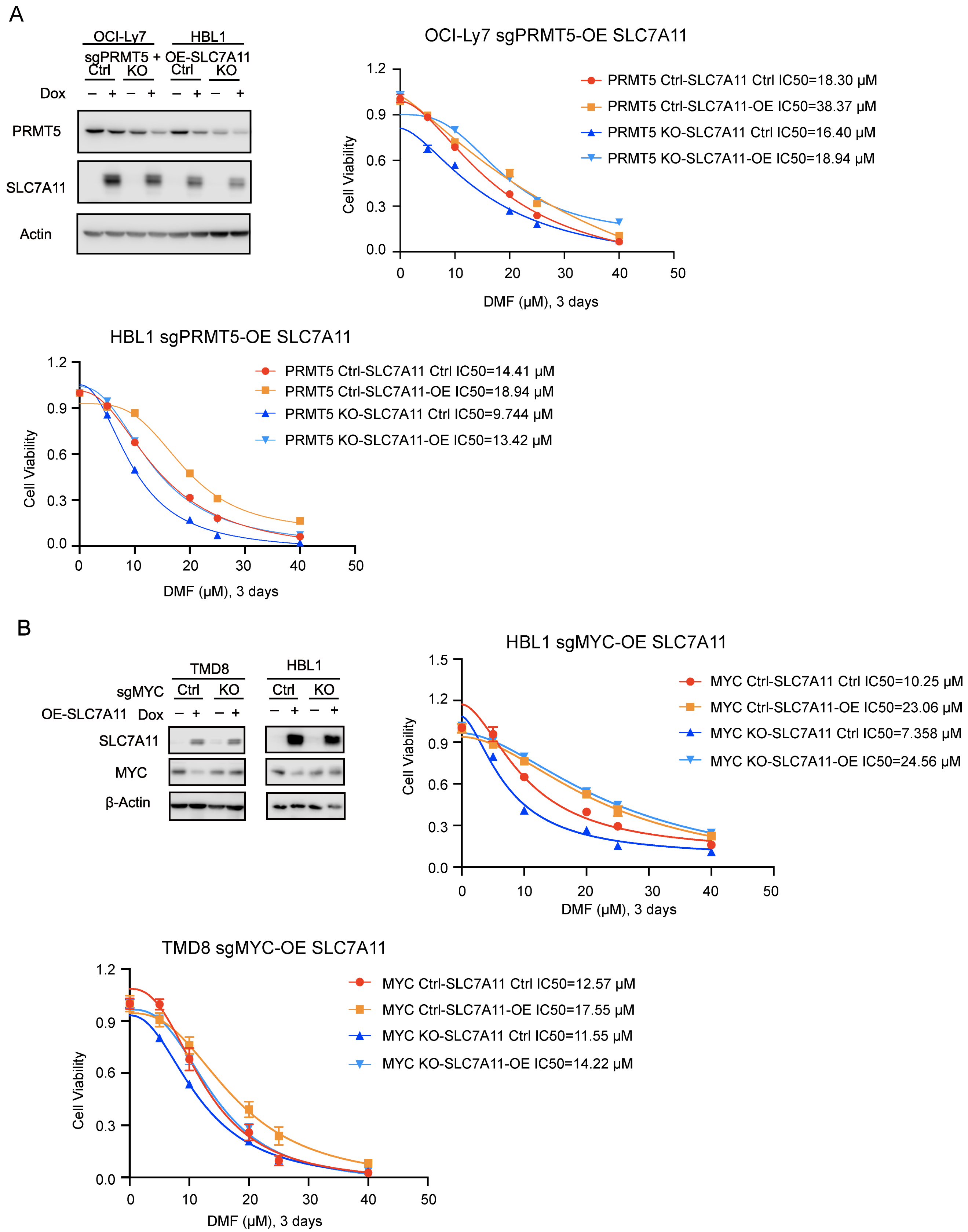

Supplemental Figure 11. SLC7A11 overexpression mitigates PRMT5 knockout- or MYC knockout–induced sensitivity to DMF. (A) Inducible overexpression of SLC7A11 using the retro-

CMV-TO-PG-SLC7A11 vector in PRMT5 knockout OCI-Ly7 and HBL1 cells reduces sensitivity to DMF, as assessed by the CellTiter-Glo™ Luminescent Cell Viability Assay. SLC7A11 expression was induced with 20 ng/mL doxycycline for 2 days, followed by 3 days of DMF treatment. Immunoblot analysis (top left) confirms efficient SLC7A11 overexpression and PRMT5 knockout in TMD8 and HBL1 cells. (**B**) Inducible overexpression of SLC7A11 in MYC knockout HBL1 and TMD8 cells similarly attenuates DMF sensitivity. Immunoblot analysis (top left) confirms SLC7A11 overexpression and MYC knockout. IC_{50} values were calculated using GraphPad Prism (v9.0) with a four-parameter nonlinear regression model.
